# Cross-lingual effects of AI-generated content on human work

**DOI:** 10.1038/s41598-025-16650-w

**Published:** 2025-08-22

**Authors:** Wesley W. Koo

**Affiliations:** Johns Hopkins University, Carey Business School, Washington, D.C., USA

**Keywords:** Languages, Artificial intelligence, Globalization of AI, Human work, Technology Equity, Information technology, Computer science

## Abstract

Artificial intelligence (AI) technologies, especially large language models (LLMs), have permeated human work around the globe, but how effective is workers’ usage and application of AI-generated content across language settings? This research examines the quality of (1) AI-generated business recommendations in English, Arabic, and Chinese and (2) business emails written by human participants in those languages. In Study 1, trained evaluators rated the quality of AI-generated content. Study 2 is a randomized experiment in which 480 human participants (160 in each language setting) wrote emails to address business issues, with or without the help of AI-generated content. Trained evaluators then rated the quality of those emails. This research finds that the AI-generated responses were of lower quality in Arabic and Chinese than in English. Importantly, human participants’ usage of AI-generated contentin email-writing was associated with less actionable and less creative work output in Arabic and Chinese than in English. Non-English participants were particularly disadvantaged when dealing with more technical work tasks (e.g., tasks related to scientific discovery or product design). These findings underscore the need for more language-inclusive LLMs to support workers worldwide, as current technologies may inadvertently widen the productivity gap between English and non-English speakers, particularly in more technical domains.

**Supplementary Information:**

The online version contains supplementary material available at 10.1038/s41598-025-16650-w.

## Global importance of AI technologies

The rapid development of artificial intelligence (AI) technologies, particularly generative AI in the form of large language models (LLMs), has changed work and human interactions^1,2^. AI-generated content has generated remarkable gains in content generation, answering questions, reasoning, computer programming, and scientific discovery^3–7^. Recent research has focused on human-AI collaboration and the variation in the effectiveness of AI adoption across populations and contexts, suggesting that AI technologies can elevate the skills of low-skilled or disadvantaged workers and allow them to compete better with high-skilled or advantaged workers^8–12^. As workers around the world begin to integrate AI into their workflow, a prevalent but understudied facet of AI in human work is the linguistic context in which the work takes place.

## Literature review

Prior literature suggests that AI technologies can help non-English speakers narrow the gap relative to English speakers in scientific work^13^, as more than 80% of the world population does not speak English. However, the most popular LLMs, such as OpenAI’s ChatGPT, Anthopic’s Claude, and Google’s Gemini, were trained primarily on English data^14,15^. Even the models of DeepSeek, a Chinese AI company, are heavily trained on English content, with its models aiming to perform well in both English and Chinese benchmarks^16^. Non-English languages, especially low-resource languages like Swahili and Khmer and medium-resource languages like Arabic and Vietnamese, face a scarcity of high-quality and diverse training data across topics and industries^17^. This disparity could result in richer, more useful, and more nuanced LLM output in English than in other languages, keeping the input/requests equal. These cross-lingual differences in output quality and quantity could lead to English speakers improving the quality and productivity of their work output more than non-English speakers using LLMs in their own languages.

Prior literature also suggests that cross-lingual differences in grammatical structure, morphology, and writing systems may engender differences in the effectiveness of AI-generated content in different language settings. In Arabic, 97% of the written text omits or does not explicitly show the diacritical marks for short vowels in words^18^. For example, without diacritical marks, the word “علم” (*eilm*) can have multiple meanings, such as “flag” and “science.” Thus, the absence of diacritical marks may make it difficult for LLMs to disambiguate the intended meaning of input text. If there are no popular, Arabic-focused models, Arabic speakers likely have to use English-focused models that produce lower-quality output in Arabic. This issue may negatively affect Arabic-speaking workers’ productivity and quality of work. Indeed, anecdotally, scholars have lamented that “in Arabic, (LLM) answers sometimes include inaccurate or nonsensical sentences.”^15^.

Current literature on the cross-lingual effectiveness of AI-generated content primarily comes from computer scientists, who have focused on benchmarking the performance of LLMs in different languages based on the completion of NLP tasks (e.g., machine translation, named entity recognition)^17,19–22^. In those studies, scholars typically employ automated metrics such as BLEU and ROUGE to calculate scores for lexical overlap and BERTScore to calculate scores for semantic similarity. They then quantify the relative performance of AI-generated content across languages by comparing these scores.

### Research motivation and overview

While prior studies provide valuable insights into the multilingual capabilities of LLMs, they usually lack a *human-centric perspective*, which focuses on identifying the effectiveness of AI-generated content in human work. Without examining human workers’ utilization of AI-generated content in different language settings, it is entirely possible that the most popular AI technologies in medium- or low-resource language settings are good enough for most work tasks. Put differently, differences found in benchmarking might not translate into differences in effectiveness. Additionally, it is unclear where (e.g., for what types of work tasks) AI-generated content create the biggest differences across languages. For instance, using the same AI-generated content in English might generate better work output than using it in Vietnamese when the task involves creating marketing materials, but not when it involves resolving customer disputes.

Therefore, given (1) a lack of training depth in non-English languages relative to in English among the world’s most popular AI technologies and (2) a lack of knowledge regarding AI’s cross-lingual effectiveness in human work, this paper asks the research question: How does using AI-generated content affect work productivity and quality across languages? It hypothesizes that human workers might benefit less (in terms of work quality and productivity) from AI-generated output in non-English settings than in English settings. In turn, workers in non-English settings may feel less confident about their work output and about AI usage than those in English settings due to a comparative lack of quality in the AI-generated input and a lack of perceived quality in work output.

To answer this question, which is of global importance, this paper conducts two studies. Study 1 (the content evaluation study) evaluates the quality of AI-generated responses to common business issues in English, Arabic, and Chinese. Study 2 (the email evaluation study) is an experiment involving three groups of human participants (English-speaking, Arabic-speaking, and Chinese-speaking) writing emails to address business issues. In Study 2, the participants write the emails with and without the help of AI-generated responses derived from Study 1. ChatGPT-4 was used to generate 600 responses to 200 typical business issues (three responses per language) in Study 1. In Study 2, 240 participants were shown an AI-generated response in one of three languages to help them write the business email, while 240 other participants were not shown any AI-generated response for the task. For the treated participants, the AI-generated responses were presented as text directly under the task instruction. SI (Supplementary Information) B1.3 provides some examples. The Methods section at the end of the paper and the SI Section A describe the methodology in detail. SI Fig. S1 presents the experimental design in a diagram. SI B3.1 and B4.1 document the demographics of the evaluators in the two studies. SI Tables S3 and S4 document the demographics of the human participants in the email-writing tasks in Study 2.

## Methods

The methodology consists of two parts. First, Study 1 evaluates AI-generated content. Specifically, ChatGPT-4 was used to generated responses to a diverse set of 200 prompts related to common business issues in marketing, customer service, human resources, and R&D. These prompts were carefully designed to cover a wide range of real-world scenarios and were translated into English, Arabic, and Chinese. ChatGPT-4 was then used to generate responses to each prompt in all three languages, resulting in 600 AI-generated responses (200 prompts × 3 languages). To evaluate the quality of these responses, a team of six native speakers (two for each language) with prior experience in the relevant business domains were recruited. The evaluators underwent a rigorous two-week training program to consistently understand the evaluation criteria, which included four dimensions: Completeness, Relevance, Actionability, and Creativity. Each evaluator assessed the 200 responses in their native language, providing ratings for the four dimensions.

Second, Study 2 evaluates human-written emails based on the AI-generated content (JHU Homewood IRB: #HIRB00018667). Specifically, a preregistered experiment was implemented, which involved 480 human participants (160 per language) proficient in English, Arabic, or Chinese. The research was performed in accordance with IRB guidelines, and informed consent was obtained from all participants. The participants were randomly assigned to either a treatment group, wherein they were provided with AI-generated content to assist their writing of an email to address a hypothetical business issue; or a control group, wherein they were instructed to write the email like they would in a typical work setting and without using any AI-generated content for assistance. For an effect size of 0.3 on a 5-point Likert scale with a standard deviation of 0.7, obtaining a type-I error rate of 0.05 with a power of 0.8 would require 68 participants in a treatment or control group. The business issues belonged to four task types: marketing, customer service, human resources, and R&D. The effects of the AI treatment on participants’ productivity (measured by email length and time taken), perceptions (confidence and perceived difficulty of their task), and email quality were compared across the three language groups. To evaluate email quality, six evaluators were trained to rate the quality of the emails along the same four dimensions as in Study 1 (i.e., Completeness, Relevance, Actionability, Creativity). They were trained to focus on the content instead of on culture- or language-specific elements such as email formats or level of politeness. SI Section B3.2 provides detailed information on the dimensions.

Simply put, a *complete* email offers a large amount of information and the underlying rationales for its recommendations; a *relevant* email is highly topical and relates to the most critical aspects of a business issue; an *actionable* email offers clear solutions toward resolving a business issue and gives examples as action steps; and a *creative* email contains multiple suggestions that lie outside the creative repertoire of the average manager. The sample includes all responses that passed the attention check and in which participants took more than the 15-minute suggested time.

SI Section A describes the research design and materials in greater detail. The experimental protocol was exempted and approved by the Johns Hopkins University Homewood Internal Review Board and pre-registered on Open Science Framework at: https://osf.io/u7s2g. The experiment was performed in accordance with relevant guidelines and regulations.

### Statistical analysis

Study 2 seeks to experimentally examine (1) AI treatment effects on outcome variables across languages and (2) the interaction effects of relevant variables (e.g., task type, AI experience). The primary outcome variables were the four dimensions of email quality: Completeness, Relevance, Actionability, and Creativity. It also examines other important outcome variables such as productivity measures (email length, time taken).

The overall treatment effect of AI assistance is estimated by the following ordinary least squares (OLS) specification:

1 $$Y_{i} = \beta _{0} + \beta _{1} AI~Treatment_{i} + \varepsilon _{i}$$

where Y_i is the rating of email i, AI Treatment_i is a binary variable indicating whether email i was written with AI assistance (1) or without (0), β_0 is the intercept, representing the average rating for emails written without AI assistance, β_1 is the coefficient of interest, capturing the treatment effect of AI assistance on email quality, and ε_i is the error term, assumed to be independently and identically distributed. This specification is employed within each language group to derive the average treatment effect (ATE) of AI assistance on outcome variables in English, Arabic, and Chinese.

To compare the level of treatment effects across language groups, the following OLS specification with language interaction terms is employed:

2 $$\begin{aligned} & Y_{i} = \beta _{0} + \beta _{1} AI~Treatment_{i} + \beta _{2} Arabic_{i} + \beta _{3} Chinese_{i} \\ & + \beta _{4} (Treatment_{i} \times Arabic_{i} )~ + \beta _{5} (Treatment_{i} \times Chinese_{i} ) + \varepsilon _{i} \\ \end{aligned}$$

where Arabic_i and Chinese_i are binary variables indicating the language of email i (English being the reference category), β_2 and β_3 capture the main effects of language on email quality, β_4 and β_5 capture the interaction effects between AI treatment and language. To investigate the language interaction effects across work types (marketing, customer service, human resources, and R&D) and other dimensions (e.g., AI experience), the above specification is adopted within subsamples. In all models, the standard errors were clustered at the participant level to account for potential within-participant correlation in email ratings.

## Results

### **Quality of AI-Generated responses across languages (Study 1)**

The first study involved trained human evaluators’ ratings of AI-generated responses across three languages (English, Arabic, and Chinese). The results reveal significant differences in response quality across languages. In SI Fig. S2 and Table S1, Arabic and Chinese show negative and statistically significant coefficients across all four dimensions (*p* < 0.001 for all eight coefficients). Compared to English responses (the constant terms represent the ratings), Arabic responses are rated 1.44, 0.36, 0.74, and 1.67 standard deviations lower in Completeness, Relevance, Actionability, and Creativity, respectively. Similarly, Chinese responses are rated 1.20, 1.74, 1.69, and 1.36 standard deviations lower than English responses along those four dimensions.

Next, this research examines the moderation effect of the type of work task (marketing, customer service, human resources, and R&D) on the relationship between languages and the quality of AI-generated responses. SI Fig. S3 and Table S2 show that, on top of the negative primary effects of Arabic and Chinese relative to English, Arabic is additionally penalized when Arabic responses pertain to the category of R&D. Specifically, the gaps between Arabic and English in Completeness (*p* = 0.005), Actionability (*p* = 0.000), and Creativity (*p* = 0.001) are larger for R&D than for the baseline task type (marketing).

### AI effects on the quality of human work across languages (Study 2)

**Fig. 1:**
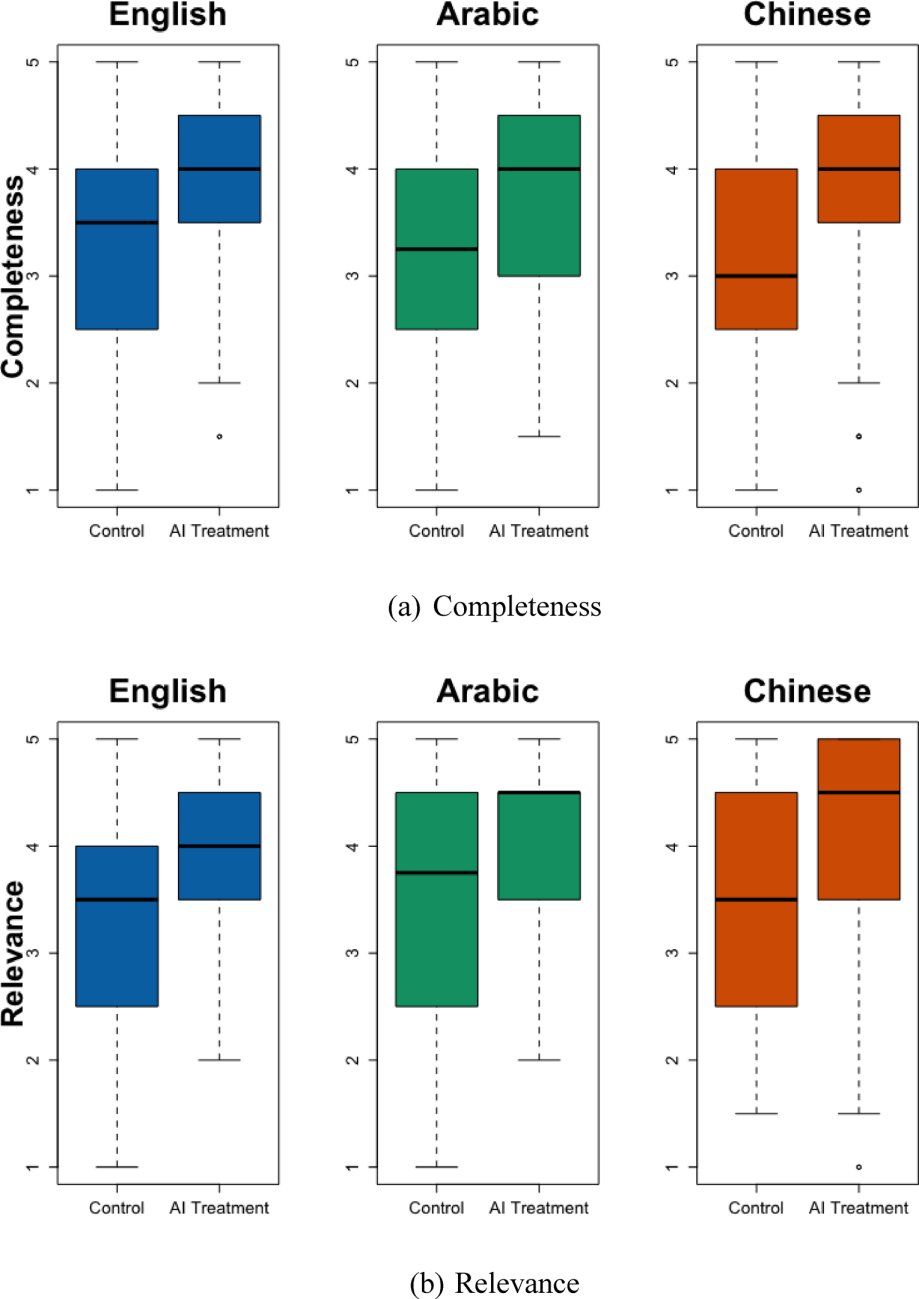

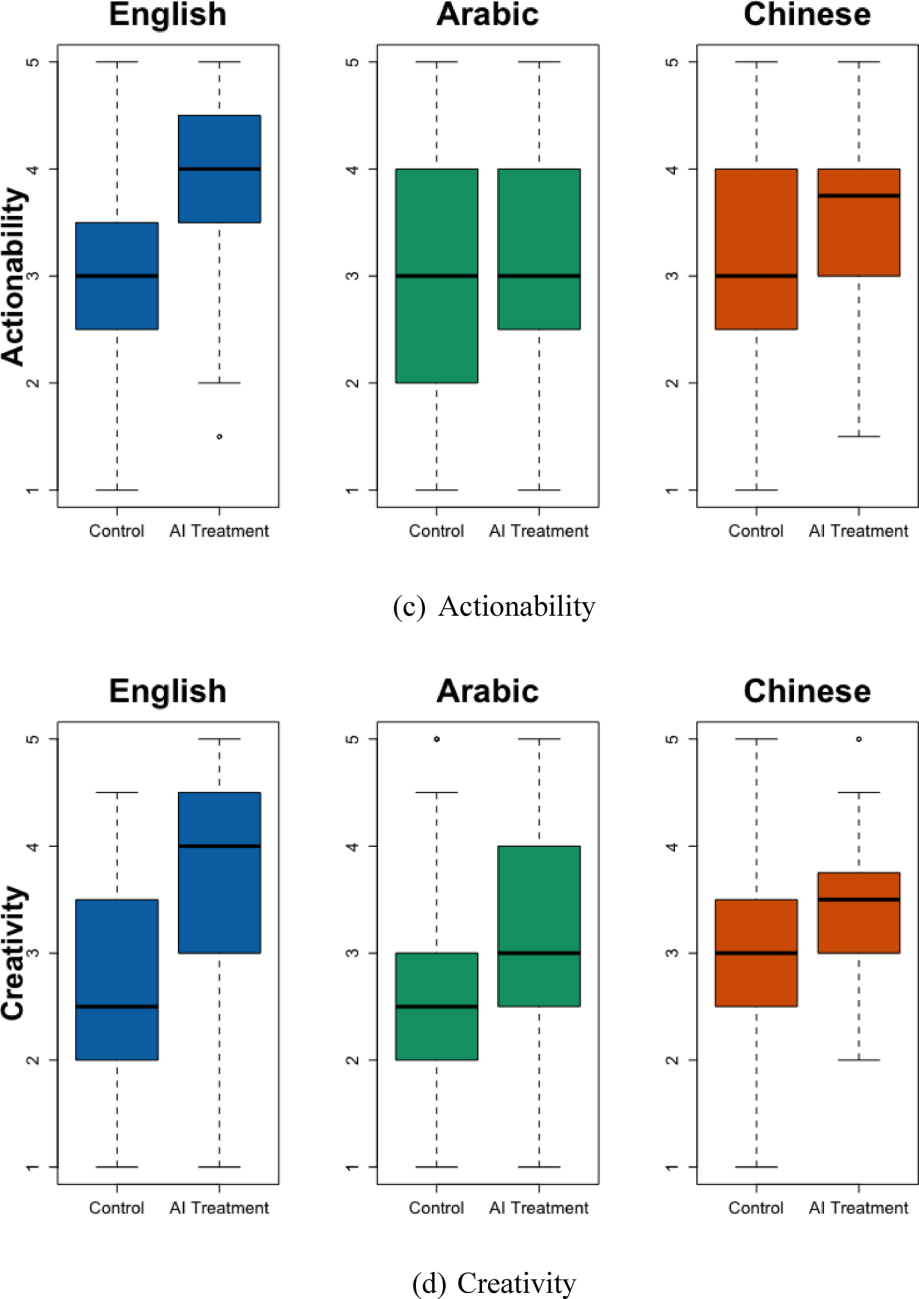
Email Quality by Language and AI Treatment. This figure shows the treatment effects of using AI-generated content by language group. From left to right, the three languages in each subplot are English, Arabic, and Chinese. The figure contains boxplots. In a boxplot, the solid black line in the middle represents the sample’s median. The box represents the interquartile range (IQR), the range between the 1st and 3rd quartiles. The hinges at the top and bottom ends are the minimum and maximum values, excluding outliers.

The second study is the core of this paper. This email-writing experiment evaluates the cross-lingual effects of providing AI-generated content (AI treatment) to human participants on the quality of the emails they wrote and their productivity and perceptions. SI Tables S3 and S4 document the descriptive statistics and balance tests. I first evaluate the email quality using the four exact dimensions as in Study 1. Figure 1 and SI Table S5 show that AI treatment led to similar levels of improvement in the Completeness and Relevance of the emails across languages. However, AI treatment is associated with 0.59 standard deviations less improvement in Actionability (*p* = 0.015) in Arabic than in English. It is also associated with 0.47 standard deviations less improvement in Actionability (*p* = 0.022) and 0.49 standard deviations less Creativity (*p* = 0.016) in Chinese than in English. SI Table S6 shows high interrater reliability between the evaluator pairs within each language group: the Kendall’s *W* values consistently reject the null hypothesis that “the evaluations show no agreement.”

While this study finds that Arabic and Chinese speakers benefit less from the AI treatment than English speakers, with the former producing less actionable and less creative emails, these results could be driven simply by an abundance of information and not specifically by AI-generated content. Since this study is primarily concerned with the language interaction effects (rather than the main effects of the AI treatment), the variation in quality outcomes across language groups needs to not be affected by the differences in the abundance of content in the AI-generated content across language groups. Information abundance is proxied by prompt length (measured as the number of normalized characters in each prompt). Within the prompts with AI treatment, Arabic and Chinese prompts are 35% and 23% shorter than English prompts. SI Table S7 shows that the triple interaction term, AI Treatment × Language × Prompt Length, is not statistically significant for most models. Next, SI Table S8, a new variable, Long A/C Prompt, is constructed to represent the longer Arabic and Chinese AI-treated prompts (measured by the ratios of Arabic to English and Chinese to English prompts). The triple interaction term, AI Treatment × Language × Long A/C Prompt, is not statistically significant in any model. These results show that the amount of information provided to the participants is unlikely to have significantly influenced the cross-lingual patterns in participants’ output quality. Instead, the quality of the AI-generated content in the prompts is a more salient determinant of participants’ output quality.

## AI effects on productivity across languages (Study 2)

This research also examines the email length (measured by the number of normalized characters) and the time to write the emails. SI Fig. S4 and Table S9 show that, whereas AI treatment led to significantly longer emails being written in all three languages, the increase in email length was uneven across languages. In English, AI treatment is associated with a 49% increase in email length (631 normalized characters; *p* = 0.000). In Arabic and Chinese, it is associated with increases of 25% (283 normalized characters; *p* = 0.010) and 46% (532 normalized characters; *p* = 0.000) in email length, respectively. The increase in email length in English is significantly greater than in Arabic (*p* = 0.034). SI Fig. S5 and Table S10 show that AI treatment is associated with a 17% increase in the time taken to write emails in English (233 s; *p* = 0.006). However, it did not lead to significant differences in the time taken to write emails in Arabic or Chinese. The increase in time taken due to AI treatment in English is significantly greater than in Arabic or Chinese.

## AI effects on human confidence across languages (Study 2)

SI Fig. S6 and Table S11 document results on participants’ self-reported confidence about their output quality and perceived difficulty of the email-writing task. AI treatment did not affect participants’ confidence in English, but is associated with 0.33 standard deviations less confidence in Arabic (*p* = 0.031) and 0.37 standard deviations more confidence in Chinese (*p* = 0.019). SI Fig. S7 and Table S12 show that AI assistance did not affect participants’ perceived difficulty of the task in English or Arabic but is associated with 0.48 standard deviations less perceived difficulty in Chinese (*p* = 0.004). These results point to cross-cultural variation in AI perceptions, with Chinese individuals more likely to embrace and feel confident about AI. I explore this further in the discussion section.

### Task types (Study 2)

**Fig. 2:**
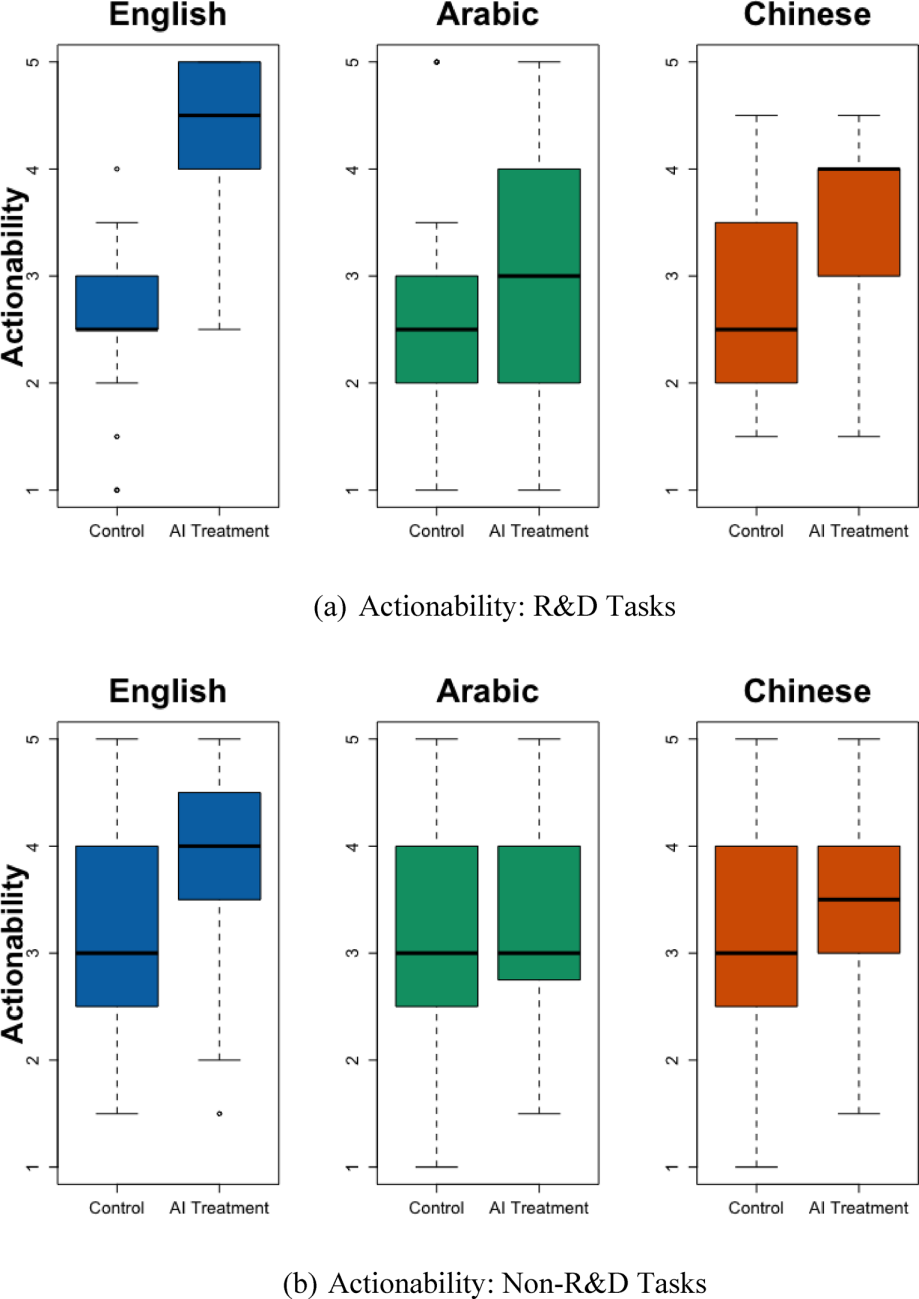
Email Actionability by Task Type (R&D vs. non-R&D). This figure shows the Actionability of the emails across languages, divided into R&D and non-R&D tasks. The subheading under Fig. 1 explains how to interpret boxplots.

**Fig. 3:**
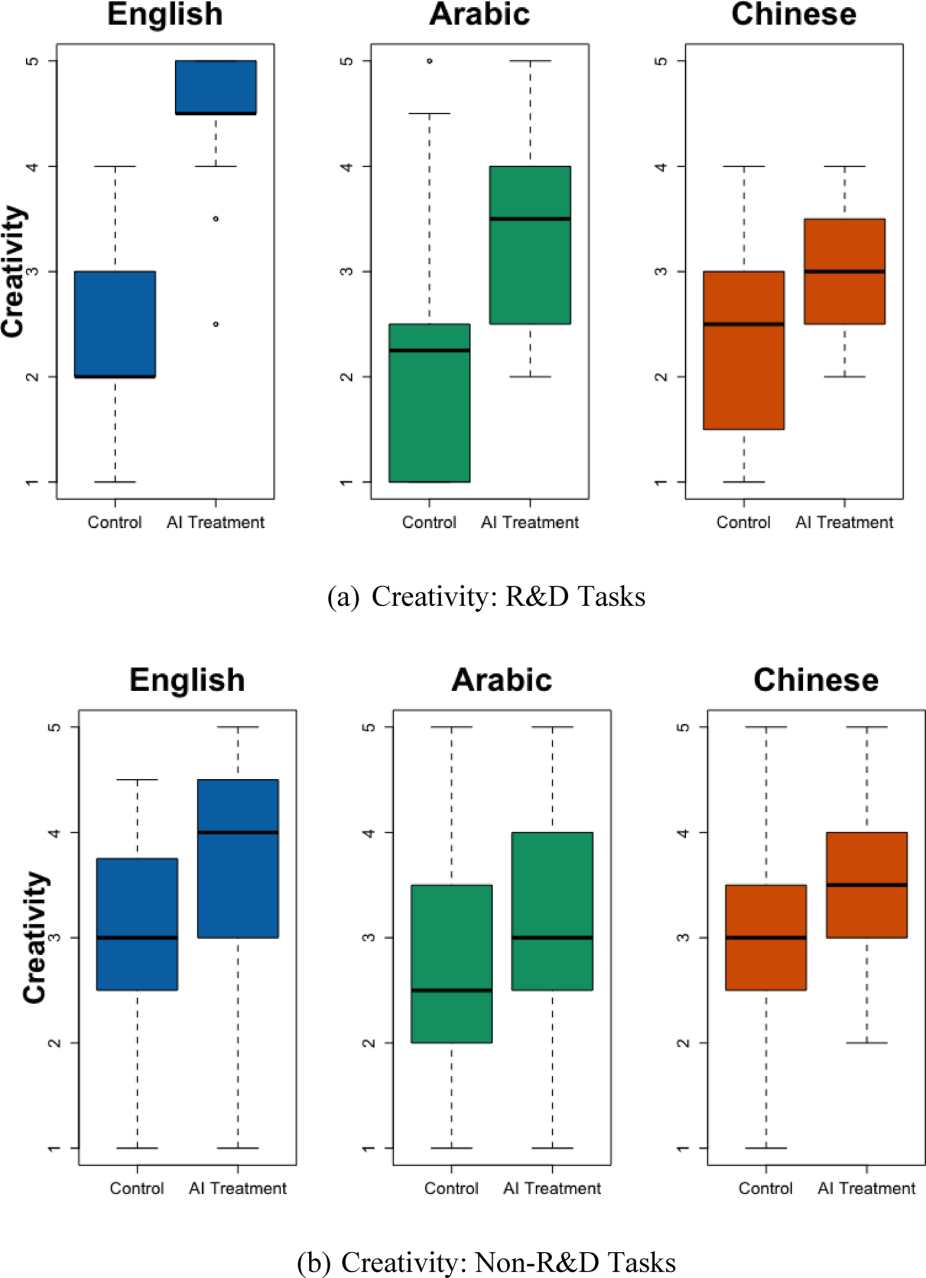
Email Creativity by Task Type (R&D vs. non-R&D). This figure shows the Creativity of the emails across languages, divided into R&D and non-R&D tasks. The subheading under Fig. 1 explains how to interpret boxplots.

This research studies how cross-lingual AI treatment effects vary across tasks. It focuses on four tasks: marketing, customer service, human resources, and R&D. The first three tasks pertain to common tasks in most business settings. The last task type (R&D) asks participants to resolve issues related to the research and development of new technologies and products—topics that are likely less known to the participants and require more AI assistance. SI Table S13 shows that, compared to participants in the baseline task type (marketing), participants who were asked to write an R&D-related email were especially likely to produce emails that were less actionable in Arabic (*p* = 0.053), less creative in Chinese (*p* = 0.025), and shorter in both languages (*p* = 0.037 and 0.020). Figures 2 and 3 separately display the language effects for R&D versus other task types.

Finally, per preregistration, heterogeneity analyses on participant demographics and experiences were conducted, producing no noteworthy results. Regressions with ordered logit models and control variables produced similar results.

## Discussion

More than 80% of the world does not speak English, but the most popular LLMs are primarily trained on data in the English language. Computer scientists and AI engineers regularly benchmark LLMs to show differential performance metrics across languages^17,20,21,23^. Still, we do not have a good understanding of the cross-lingual effectiveness of LLMs in human work and productivity^13^. This research evaluates both AI-generated responses and human-written emails across English, Arabic, and Chinese. This research documents four noteworthy findings. First, AI-generated content in Arabic and Chinese was rated as less complete, relevant, actionable, and creative than English content. Second, relative to English, providing AI-generated content to human participants in Arabic and Chinese was associated with less actionable and less creative work output (i.e., emails they wrote to address work-related issues). Third, there was significant cross-lingual variation in how participants perceived and trusted AI. Chinese speakers were substantially more confident about their AI-assisted work than English or Arabic speakers. Fourth, the evaluations of both AI-generated content and human-written emails show that ChatGPT-4 fared especially poorly in Arabic and Chinese relative to English when it involved R&D tasks (e.g., development of better product technologies).

Collectively, these findings suggest that, during one-off interactions between users and AI, using AI-generated content may lead to significantly different levels of output quality and productivity across languages. Although companies in non-English-speaking countries have developed their own LLMs (e.g., Qwen in China and Jais in the UAE), most non-English speakers tend to use English-based products, including ChatGPT, Claude, and Midjourney, even for tasks in their languages. For instance, a great majority of Arabic-speaking LLM users choose to use ChatGPT over Arabic-focused products^23,24^. When AI-generated content is less actionable or creative in non-English languages, it may hinder non-English speakers’ ability to fully capture AI’s productivity benefits compared to English speakers. While AI is touted to have the potential to elevate workers in less advantaged positions (e.g., low-skilled workers)^25,26^, this research shows that it could reinforce the dominant position of advantaged populations.

The finding on the cross-lingual variation in AI perceptions and confidence is also noteworthy. It points to potential cultural differences in the acceptance and embrace of LLM, with Chinese individuals displaying more positive attitudes. This observation aligns with recent surveys highlighting that people in eastern countries are, on average, more optimistic and confident about AI usage^27^. However, in light of the finding that AI treatment was associated with lower-quality emails in Chinese than in English, I caution that more confidence in AI does not necessarily lead to better work with AI-generated content. This result implies that there might be a stronger drive to use LLMs among Chinese speakers, leading to more but potentially worse AI-assisted work output than among speakers of other languages.

Additionally, it is notable that R&D-related emails were rated particularly low with AI treatment regarding its Actionability and Creativity. This result corresponds to the evaluation of AI-generated content, which shows that R&D-related content was particularly low-quality in non-English languages. Prior work in computer science indicates that a lack of training data on more technical and esoteric topics in non-English languages may have led to this result^17,19^. The practical implication of the R&D result is that the usage and deployment of LLMs must consider the specific requirements of different work contexts across languages, with particular attention devoted to technical work tasks in non-English languages. The attention to technical work tasks is crucial because those tasks, which underlie scientific discovery and product development, are key determinants of economic growth and advancement in businesses, communities, and countries.

This study contributes to understanding the cross-lingual effectiveness of AI-generated content in human work. By highlighting the disparities in AI performance and its impact on human productivity and perceptions across English, Arabic, and Chinese, this study underscores the need for more inclusive and equitable AI solutions that cater to the diverse needs of global workforces. As LLMs continue to transform the nature of work, it is crucial—for workers, AI companies, and society—to be keenly aware of the cross-lingual limitations of these technologies.

As AI translation becomes increasingly seamless^28^, one may argue that English AI output can simply be automatically translated into workers’ respective languages. However, AI output generated from training on English content might not apply to other language contexts, variation of which is highly correlated with variation in business standards, culture, and guidelines of social interaction. Therefore, AI developers should consider expending more effort in training and fine-tuning their models for low- and medium-resource languages. For example, developers may create tools that utilize targeted prompt engineering and retrieval augmented generation (RAG) to account for linguistic and contextual heterogeneity^29^. Developers may also consider using scaling laws to optimize data allocation in low-resource language settings^30^. It might be necessary to create certain types of content (e.g., scientific materials, historically significant documents) in low-resource languages for training and finetuning. In addition to these techniques, however, a human-centric examination of the trained models, which could involve human participants’ usage of AI technologies in both experimental (such as Study 2 in this research) and real-world settings, would be an essential building block toward improving AI effectiveness across languages.

## Conclusion

This research takes a human-centric approach and reveals critical disparities in the cross-lingual effectiveness of applying AI-generated content to human work tasks. Through two studies evaluating AI-generated responses (Study 1) and their impact on human work output (Study 2), this research demonstrates that non-English speakers experience diminished productivity gains: AI assistance in Arabic and Chinese led to less actionable and creative emails, with those disadvantages becoming especially pronounced in technical tasks like R&D. These findings underscore two key contributions. First, while many around the world are championing the potential of AI to level the playing field for less advantaged worker populations, this research exposes how cross-lingual differences in AI utilization may exacerbate productivity gaps between human work, especially in terms of the actionability and creativity in work tasks. Second, it calls for linguistically inclusive AI models to enhance the distribution of AI-driven benefits in workplaces around the globe, especially related to more technical work tasks. These contributions contain practical implications for workers, employers, policymakers, model engineers and designers, and the digital companies and platforms in charge of distributing the technology.

### Limitations

This study is limited in several ways. First, since the participants were spread across the world and may not have access to the same LLMs, treated participants in Study 2 were presented pre-generated LLM responses instead of interacting with an LLM in repeated and more elaborate fashions (e.g., prompt-engineering, building custom agents). Thus, the experimental results should be interpreted as the effects of providing AI-generated content on work output in single, interactions. Second, this study relied on content generated from one LLM (GPT-4). Rapid advancements in LLMs and AI agents, coupled with their increased integration into work routines, may generate cross-lingual comparisons that are different from that uncovered in this study. Third, despite the randomization and balance tests using control variables, this paper cannot rule out the possibility that the observed differences are driven by inherent differences in AI-specific capabilities or characteristics across the English-, Arabic-, and Chinese-speaking populations (e.g., trust in AI). Even though the calibration training tried to ensure that the evaluators would produce similar ratings for similar-quality outputs in different languages, variation in evaluators’ industry backgrounds and education might still unintentionally affect their rating standards. Fourth, whereas the training asked evaluators to think about cultural appropriateness in the Actionability dimension, cultural differences across language settings can also impact other dimensions like Creativity. Future work may experimentally manipulate the cultural environment in which users use AI to tease apart the effects of socio-cultural and more technical factors. Evaluations of those dimensions did not account for cultural differences. Finally, the sample of human participants in Study 2 may lack representativeness, as they are younger (average age of 31) and likely more tech-savvy than the general population. These characteristics may generate estimates that are different from the population-wide effects.

## Supplementary Information

Below is the link to the electronic supplementary material.

Supplementary Material 1

## Data Availability

Evaluation data can be accessed at: https://figshare.com/s/b27991ce8ea36143d7ef.
