# Supplementary material for: Cross-lingual effects of AI-generated content on human work
Source: Sci Rep. 2025 Aug 22;15:30949. doi: 10.1038/s41598-025-16650-w (PMC12373962; doi:10.1038/s41598-025-16650-w)
Supplement: Supplementary file 1 — Supplementary Material 1 [file 41598_2025_16650_MOESM1_ESM.docx]

**Supplementary Information**

**Cross-Lingual Effects of AI-Generated Content on Human Work**

**Supporting Text**

1. **Detailed Information on Research Design**

**A1. Experimental Methodology (Study 2)**

Section B2 contains the preregistration document. The sections below document the participants, the recruitment process, and survey structure. They also detail the email-writing task, evaluation process, and model specifications.

**A1.1. Participants**

The experiment in Study 2 involved a sample of 480 participants, consisting of 160 native speakers each from three language groups: English, Arabic, and Chinese. The study recruited the participants from Prolific, an online human-subject research platform with a global representation of participants (Douglas, Ewell, & Brauer 2023). Participants were selected based on their language proficiency (being able to speak a given language fluently). The study also required participants to be at least 18 years old.

**A1.2. Recruitment**

Three tasks were set up on the platform. Participants were invited to complete an email-writing task in a given language (English, Arabic, or Chinese). They were informed that the task would take approximately 20 minutes and that they would be paid 4 USD for completed tasks. Data collection started on March 21 and lasted till March 27, 2024, which is in line with preregistered recruitment criterion (tasks kept open for one month or having collected 160 responses per language group, whichever comes earlier). At the time of the experiment, the available participant pool for the English group (124,923) is substantially larger than that for Arabic (939) or Chinese (2,044). The study also rejected nine Arabic responses and six Chinese responses because their responses were adjudicated to be irrelevant to one or more survey questions. These rejected responses are not a part of the final sample.

**A1.3. Survey Structure**

480 screened-in participants (160 per language group) were taken to one of three surveys on Qualtrics. The English survey is provided in Section B1. Section B1.1 provides screenshots of how the survey appeared in three languages. Participants were first required to read the consent form and provide informed consent before proceeding to the next section. Then, participants answered an attention check question on the payment scheme (consent form available upon request). The study included those who failed the attention check questions in the 480-participant sample, but excluded them during the analysis. Next, participants were asked to complete an email-writing task (more information in the next section). This is the core part of the survey. Subsequently, participants would answer questions on how often they use AI tools in work or life, how difficult they found the email-writing task, and how confident they were about the quality of their email. Then, they were asked to write two to sentences about how they felt about the task. Finally, they were directed back to the platform for payment.

From the platform, demographic information including age, gender, ethnicity, country of birth, country of residence, fluent languages, first language, nationality, and employment status were also collected. Qualtrics also recorded the time taken to complete the survey and the number of clicks.

**A1.4. Email-Writing Task**

Within each language group, each participant was randomly shown one of 16 scenarios. In each scenario, participants were presented with a business issue at hypothetical company or organization. The scenarios were designed to mimic real-world business challenges. Participants were required to write an email to their colleagues at the hypothetical company to address the business issue.

Eight of the 16 scenarios contained AI-generated responses to help with the task (AI treatment), and the other eight scenarios contained just descriptions of the business issue (control). In a treatment scenario, the AI-generated content was created using OpenAI’s ChatGPT-4, which was employed to generate responses to the business issues in the 16 scenarios. In a control scenario, participants wrote their email responses without any AI assistance. They were provided with the same hypothetical scenario as the corresponding treatment scenario but were asked to rely solely on their own expertise and explicitly asked to not use AI tools.

The study also varies the 16 scenarios by the type of tasks: four scenarios (two in treatment, two in control) asked participants to address issues in Marketing; four in Customer Service; four in Human Resources; and four in R&D. In all scenarios, participants would write their emails in a textbox below the prompt. They were reminded by a line of red text that they should spend at least 15 minutes on the task. A clock ticker at the end of the page showed how much time had elapsed since they started the writing task.

The scenarios were composed to reflect managerial tasks and issues that are highly common in business settings around the world. For example, a scenario relates to developing a software app to promote financial literacy and another scenario relates to creating wellness programs to improve employees’ mental health. For R&D task type, the study included industry-specific issues but asked participants to address the issues from a business perspective. Section B1.2 provides some sample text of the scenarios in English.

In an ideal world, the study would make participants in the treatment group write the email while interacting with LLMs and participants in the control group write the email without LLMs. However, the pilot interviews with workers in the U.S., China, Egypt, Lebanon, and Saudi Arabia revealed several factors that made this ideal design impractical. First, given the highly global nature of this study, it was necessary to ensure that all participants have similar levels of access to LLMs. The interviews revealed that China still officially blocked LLMs including ChatGPT and Claude at the time of this study, although a substantial number of working professionals (e.g., software engineers, marketing employees, ) were using ChatGPT through VPNs. Also, two Egyptian correspondents told us that, in Egypt, they needed a foreign phone number to access ChatGPT, or people needed to register third-party platforms such as Poe (https://poe.com/) to use services offered by ChatGPT and Claude. Second, while in theory the study could have included different LLMs in the experiment, it would be exceedingly difficult to control for the LLM-specific factors in the analysis. Most of these LLMs are not open-source, which disallows us from gaining a deep understanding of how two LLMs are different or similar to each other. For example, if the Chinese-speaking participants used Wenxin (a popular LLM in China) but the English-speaking and Arabic-speaking participants used ChatGPT, it would be difficult for us to parse out the differences between Wenxin and ChatGPT, which is essential for being able to attributing any outcome difference to LLM type versus cross-lingual differences within an LLM. Third, the study also explored the possibility of recruiting participants offline and conducting the experiment in a lab setting or a field experiment. However, it became apparent that the study could not reach a good enough sample size, as it was difficult to find enough Arabic-speaking and Chinese-speaking participants in metropolitan area of the PI’s home institution. Due to these reasons, the study opted to conduct the experiment in a controlled environment by focusing on online participants’ exposure to AI-generated content rather than making them write the emails through interactions with LLMs.

**A1.5. Evaluation**

Six evaluators who are native speakers of English, Arabic, and Chinese (two in each language group) were recruited to evaluate the quality of the email responses. The evaluators were recruited via the job platform at a major private research university on the east coast of the United States. The university has a wide body of international students and students of diverse backgrounds. Candidates who submitted their resumes were screened for managerial experience with at least one year of management-related work experience at companies, organizations, or government entities. Section B3.1 contains relevant information about the evaluators.

Each email response was independently evaluated by two evaluators who were native speakers of the language in which an email was written. The evaluators rated the emails with a predetermined set of criteria along four dimensions: Completeness, Relevance, Actionability, and Creativity. Section B3.2 provides detailed information on the evaluation criteria for each dimension. The decision to evaluate the emails on these four dimensions was rooted in the management literature on knowledge and absorptive capacity, which emphasized the importance for workers to acquire, assimilate, transform, and apply a piece of information or knowledge ^1,2^. Completeness and relevance relate to workers’ ability to acquire and assimilate knowledge into their work. Actionability relates to the process of transforming and applying knowledge to the work tasks at hand. Creativity relates to the rarity and non-imitability of knowledge.

To evaluate *Completeness*, the criteria focus on the presence of two features in an email: 1) the amount of information (e.g., the number of suggestions and bullet points) and 2) whether it provides ample rationales for the suggestions. To evaluate *Relevance*, the criteria focus on the degree to which an email caters to the key parts of the prompt/business issue. For example, if a business issue is about improving retention at a healthcare facility, the email should provide suggestions that revolves around not only employee retention, but also the fact that it is a healthcare facility (i.e., not other types of facilities). To evaluate Actionability, the criteria focus on the presence of four features in an email: 1) clear action steps, 2) ample examples to illustrate the action steps, 3) whether one can interact with an email by building on it and engaging in further learning and 4) whether an email is culturally appropriate. To evaluate Creativity, the study asked evaluators to think from the perspective of the average manager and decide if an email contains one or more suggestions that constitute “thinking outside the box” for the average manager.

Each dimension was rated on a 5-point scale, with 1 being the lowest (poor rating) and 5 being the highest (excellent rating). The evaluators were aware of the goal of this study (i.e., to examine AI effects on work quality and productivity across languages), but they were blinded to an email’s treatment status (AI assistance versus no AI assistance). After providing four ratings along the four dimensions, evaluators also gave brief comments on the rationales underlying their ratings.

For the purpose of this study, it is of utmost importance to ensure that similar criteria are applied when rating emails written in different languages. To do that, the evaluators participated in rigorous training over a two-week period prior to rating the email responses. In the first week, the study engaged all evaluators in two live training sessions that introduced the rating criteria and conducted four exercises. In each exercise, the study asked them to rate an email and tell us their ratings and rationales. Then, the study revealed the “correct” ratings as determined by the criteria outlined above and discussed why their ratings were too high or too low. Section B3.3 provides sample training exercises.

In the second week of training, the study sent the evaluators two calibration exercises via email. The goal of the calibration exercises was to ensure that they could independently apply the evaluation criteria and produce the correct ratings and similar ratings as others rating the same emails in a different language. The evaluators sent us their ratings via email, and we calibrated their ratings by explaining to them whether and why their ratings were too high or too low, also via email. Section B3.4 provides a sample calibration exercise and sample exchanges between the principal investigator and the evaluators.

The final ratings for each email response were calculated by averaging the ratings provided by the two evaluators within a language group along each dimension. Table S13 documents the Kendall’s *W* coefficients of concordance between the evaluators within each language group. *W* = 0 indicates no agreement, and *W =* 1 indicates perfect agreement between evaluators ^3^. It also documents the associated p-values in the same table. A low p-value indicates increased likelihood of rejecting the null hypothesis of “no agreement among evaluators.” ^4^ In other words, a statistically significant p-value indicates a high likelihood of agreement among evaluators. The Kendall’s *W* coefficients generated by the evaluators’ ratings are high in each dimension and for each evaluator pair. They range from 0.71 to 0.88 (all p-values < 0.001), indicating high level of agreement between pairs of evaluators within each language group.

**A2. AI Content Evaluation (Study 1)**

The evaluation of AI-generated responses to address business issues happened before the experiment. The evaluation criteria and training process for evaluating AI-generated responses are similar to those for evaluating the human-written emails from the experiment. Section B4.2 provides the criteria for AI content evaluation. The central pillars in the evaluation criteria (e.g., the four core features of Actionability) are exactly the same as those in the criteria for evaluating the emails. Similar to the experiment, the study recruited six evaluators who are native speakers of English, Arabic, and Chinese. They also went through two weeks of training, involving both live training sessions and calibration exercises. The evaluators were master’s students with at least one year of management-related work experience and were selected via the university’s job platform.

1. **Materials**

**B1. Key Information from the Experiment**

**B1.1. Email-Writing Task: Sample Screenshots**

Below are sample screenshots of the email-writing task in English, Arabic, and Chinese.

**English task**


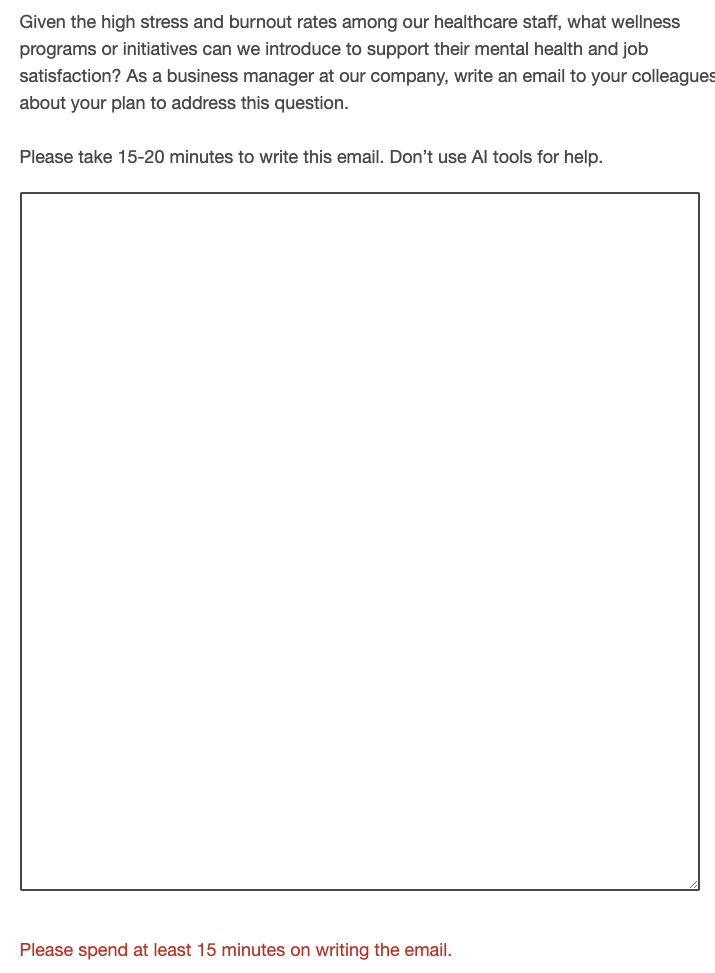


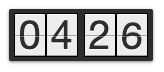


**Arabic task**


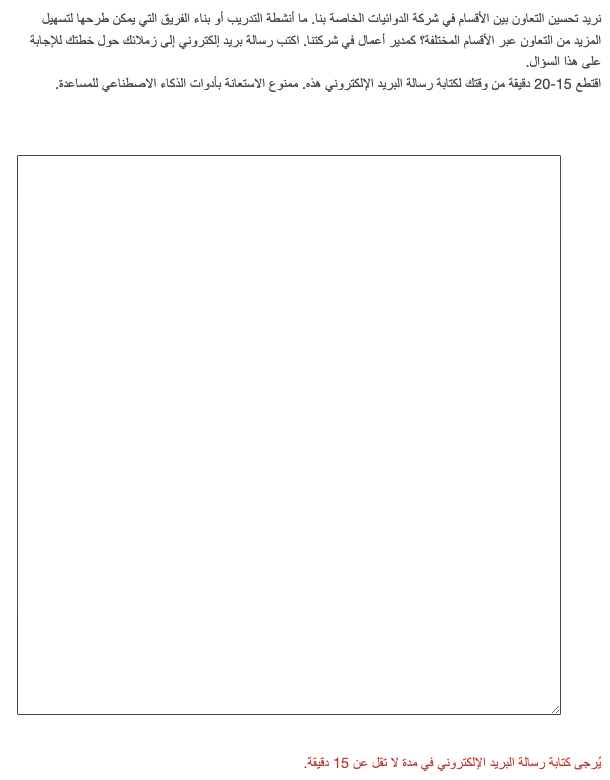


**
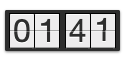
**

**Chinese task**

This task screen is longer because it contains an AI-generated response to help participants write the email (the italic text).

**
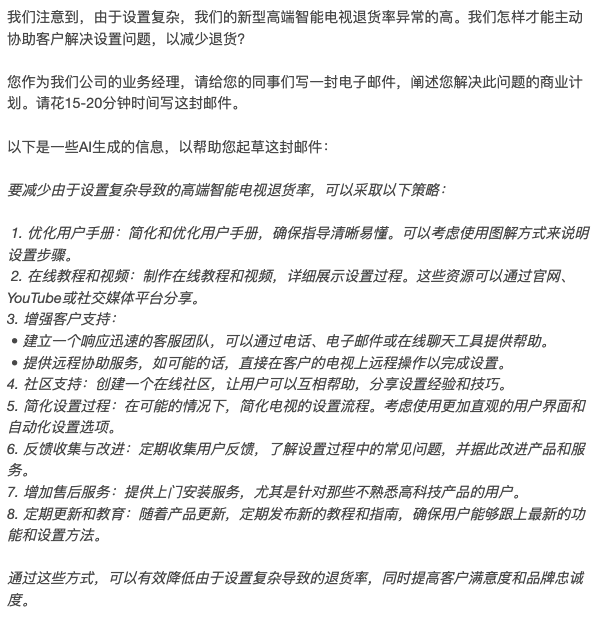
**

**
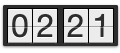
**

**B1.2. Email Scenarios**

In this section, we present the English text of two of the 16 scenarios into which participants were randomly assigned. For the rest of the scenarios, we document the scenario names only so as not to make the SI too long. Other scenario texts are available upon request. Each scenario was professionally translated into Arabic and Chinese and presented to participants in those two language groups. Here, we use blue font to differentiate the scenario text from the other text; in the survey, all scenarios had black font.

Scenario #1: No AI assistance, Marketing issue

We are a software development company. We have developed an app that promotes financial literacy, aiming to target low-income neighborhoods where this knowledge is crucial yet scarce. How should we tailor our marketing approach to be effective and sensitive to the needs of this demographic? As a business manager at our company, write an email to your colleagues about your plan to address this question.

Please take 15-20 minutes to write this email. Don’t use AI tools for help.

Scenario #2: With AI assistance, Marketing issue

We are a software development company. We have developed an app that promotes financial literacy, aiming to target low-income neighborhoods where this knowledge is crucial yet scarce. How should we tailor our marketing approach to be effective and sensitive to the needs of this demographic? As a business manager at our company, write an email to your colleagues about your plan to address this question.

Please take 15-20 minutes to write this email.

The following is some AI-generated information to help you draft the email:

*Marketing an app that promotes financial literacy, especially targeting low-income neighborhoods, requires a thoughtful and nuanced approach. Here are key strategies to consider:*

- *Understand the Audience: Research the specific needs and challenges faced by residents in low-income neighborhoods. Understand their financial situations, including common financial issues and goals.*
- *Inclusive and Relatable Messaging: Use language and imagery that resonate with the community. Avoid jargon or overly technical terms. The messaging should be relatable, showing that you understand their financial challenges and aspirations.*
- *Leverage Community Networks and Influencers: Collaborate with local community leaders, groups, and influencers who already have the trust of the neighborhood. They can be instrumental in spreading the word and endorsing the app.*
- *Educational Content: Create educational content that not only promotes the app but also provides valuable financial knowledge. This can include blog posts, videos, or workshops on budgeting, saving, credit management, etc.*
- *Accessible and Mobile-Friendly: Ensure that your marketing materials and the app itself are accessible on mobile devices and easy to navigate. Many in low-income areas may primarily access the internet via smartphones.*
- *Use of Social Media and Digital Platforms: Utilize social media platforms that are popular in these communities. Tailor your content to be shareable and engaging on these platforms.*
- *Success Stories and Testimonials: Share real stories of people from similar backgrounds who have benefited from the app. This can create a personal connection and demonstrate the app's effectiveness.*
- *Partnerships with Local Businesses and Organizations: Partner with local businesses, schools, and non-profits to promote the app. They can help distribute flyers, place ads, or even incorporate the app into their services.*
- *Affordability and Transparency: Emphasize any free aspects of the app or affordable pricing structures. Be transparent about any costs or premium features.*
- *Feedback and Adaptation: Regularly seek feedback from users and adapt your approach accordingly. This shows that you are committed to serving their needs and continuously improving the product.*
- *Multilingual Support: If the community is diverse, consider offering marketing materials and app features in multiple languages.*

*Remember, the goal is not only to market the app but also to genuinely assist in improving financial literacy, which builds long-term trust and credibility.*

Scenario #3: No AI assistance, Marketing issue

Scenario #4: With AI assistance, Marketing issue

Scenario #5: No AI assistance, Customer Service issue

Scenario #6: With AI assistance, Customer Service issue

Scenario #7: No AI assistance, Customer Service issue

Scenario #8: With AI assistance, Customer Service issue

Scenario #9: No AI assistance, Human Resources issue

Scenario #10: With AI assistance, Human Resources issue

Scenario #11: No AI assistance, Human Resources issue

Scenario #12: With AI assistance, Human Resources issue

Scenario #13: No AI assistance, R&D issue

Scenario #14: With AI assistance, R&D issue

Scenario #15: No AI assistance, R&D issue

Scenario #16: With AI assistance, R&D issue

**B2. Preregistration**

Below we present the preregistration document. The experiment was preregistered on Open Science Framework before data collection ensued. OSF registration number is hidden during double blind review.

**Study Information**

Research Question: How does the use of AI affect the quality of written communication across different languages (English, Arabic, and Chinese)?

**Hypothesis:**

The effectiveness of AI usage in written communication varies across languages.

That effect varies for different performance dimensions.

**Design Plan**

**Study Type:**

Experiment: This study will be an online experiment involving human participants, with treatments randomly assigned. It classifies as an intervention experiment.

**Blinding:**

Participants are unaware of the specific hypothesis being tested and the existence of different treatment conditions. We will provide participants with a consent form that describes the project in general terms without revealing the exact nature of the AI assistance or the comparison between languages. The trained evaluators who assess the quality of the written communication are blinded to the participants’ assigned conditions (treatment or control).

**Study Design:**

Between-subjects design with six groups (2x3) based on condition (treatment, control) and language (English, Arabic, Chinese). They will be tasked with writing an email in a managerial context about their plan to address an existing business issue. Treated participants will see AI-generated content to help with the writing task.

**Randomization:**

We will randomly assign 480 participants (160 per language group) to either the treatment or control condition. Each language group will be evenly split, with 80 participants in the treatment group and 80 in the control group.

**Sampling Plan**

**Data Collection Procedures:**

Participants will be recruited online via platforms like Prolific, targeting individuals fluent in English, Arabic, or Chinese.

**Sample Size:**

We aim to collect 480 responses, with 160 in each language group. Within each language group, we aim to collect 80 in the treatment group and 80 in the control group. We will stop collection in any language group when we have 160 responses in that group or after 1 month of data collection, whichever condition is met first.

**Sample Size Rationale:**

When dependent variables are measured on a 5-point Likert scale, an effect size of 0.3 (e.g., a difference between a 3.7 in control and 4.0 in treatment) with a standard deviation of 0.7, a type-I error rate of 0.05, and a power of 0.8 would require 68 participants in a treatment or control group. We aim to recruit 80 participants in each group.

**Variables**

- AI Treatment: The usage of AI-generated answers to assist in writing their business plan.
- Language Groups: participants who are fluent in English, Chinese (standard mandarin), or Arabic (modern standard Arabic).
- Primary Outcome Variables: The quality of the business plans, as assessed by trained evaluators using a set of predefined criteria (completeness, relevance, actionability, and creativity). Participants’ reactions to the task (e.g., perceived difficulty).
- Heterogeneity: Participants’ demographics, AI experience, and managerial experience.

**Analysis Plan**

Linear regressions and means comparison tests will be conducted to assess the main effects and interactions between the treatment and language groups on the quality of participants’ written communication. We will also carry out heterogeneity analysis to explore the differential patterns based on demographics, managerial experience, and self-reported measures. We will analyze the relationships with and without control variables. Alternative specifications will serve as additional analysis.

**B3. Email Evaluation Materials**

**B3.1. Email Evaluators’ Backgrounds**

|  | *Evaluator* | *Gender* | *Program* | *Work Experience* | *Industry* | *Country of Origin* |
| --- | --- | --- | --- | --- | --- | --- |
| English | Evaluator 1 | M | Engineering | 5 years | Software,  Manufacturing | India |
|  | Evaluator 2 | F | Informatics | 2 years | Pharmaceuticals,  Healthcare | India |
| Arabic | Evaluator 3 | F | Biology | 1 year | Government | Saudi Arabia |
|  | Evaluator 4 | M | Int’l Studies | 4 years | Culture, Government,  Construction | Saudi Arabia |
| Chinese | Evaluator 5 | M | Economics | 1 year | Software,  Government | China |
|  | Evaluator 6 | F | Finance | 2 years | Finance,  Education | China |

**B3.2. Email Evaluation Criteria**

**Completeness:**

- 5 (Excellent): The email thoroughly addresses all aspects of the given business issue, providing a comprehensive and detailed response. It offers rich information on multiple aspects of the issue and explains the rationales for its proposed solutions.

- 4 (Good): The email covers most aspects of the business issue in detail and provides some explanations for its recommendations. It offers a nearly complete response, though there may be minor areas that could be further enriched and elaborated upon.

- 3 (Average): The email addresses the main points of the business issue but lacks depth, information, and examples in certain areas. Some aspects are not fully explored, leaving room for additional information or clarification.

- 2 (Below Average): The email addresses only a few elements of the business issue, leaving significant gaps in the information provided. It lacks depth and breadth, failing to cover important aspects of the issue or give good examples. It provides little explanation for its recommendations.

- 1 (Poor): The email barely addresses the business issue, missing key components and providing a very superficial overview. It leaves most of the issue unresolved and fails to provide a useful understanding of the topic. It gives no explanation for its recommendations.

**Relevance:**

- 5 (Excellent): The email directly addresses the given business issue with highly focused and pertinent information. It stays on topic throughout, and all information provided is clearly related to the issue, enhancing the reader's understanding of the subject.

- 4 (Good): The email is mostly focused on the business issue, with most information being relevant and topical. There may be minor digressions, but they do not significantly detract from the relevance of the response.

- 3 (Average): The email addresses the business issue but includes some irrelevant or tangential information. While the core of the response is relevant, there are noticeable deviations from the main topic.

- 2 (Below Average): The email only partially addresses the business issue, with much of the information being irrelevant or off-topic. It frequently deviates from the issue and fails to stay focused on the main subject.

- 1 (Poor): The email largely fails to address the business issue, consisting mainly of irrelevant or unrelated information. It does not provide a meaningful or relevant response to the issue.

**Actionability:**

- 5 (Excellent): The email provides highly actionable and practical solutions to the business issue. It offers clear action steps or strategies that can be easily implemented. It gives rich examples that illustrate how its recommendations can be put into practice and inspires future interactions and learning at the company. It also has a high level of cultural appropriateness.

- 4 (Good): The email provides actionable solutions to the business issue, with practical advice and examples. It offers clear action steps or strategies that are generally easy to implement, though there may be minor areas lacking in practicality. It opens doors to future interactions and learning at the company. It is also culturally appropriate.

- 3 (Average): The email offers some actionable solutions, but they may be somewhat vague or lacking in illustrations. While it provides a direction or approach, more specific details or steps may be needed for effective implementation. It might or might not be culturally appropriate.

- 2 (Below Average): The email offers limited actionable solutions, with recommendations that are difficult to implement. It lacks practicality and illustrations, and it fails to provide clear or detailed action steps. It is difficult for colleagues to engage with the email and pursue further learning. It might lack in cultural appropriateness.

- 1 (Poor): The email provides no actionable solutions to the business issue. It offers no clear recommendations or examples, leaving the issue unresolved and failing to address practical implementation. It leaves little to no room for further engagement from colleagues. It might contain suggestions that are highly inappropriate given the cultural context.

**Creativity:**

- 5 (Excellent): The email demonstrates exceptional creativity, offering unique, innovative ideas or solutions. It shows original thinking and presents concepts or examples that are novel and thought-provoking. It contains multiple examples or suggestions that lie outside the average manager’s creative repertoire.

- 4 (Good): The email is creative, providing fresh insights or approaches that go beyond conventional thinking. It offers new ways of addressing the business issue and includes non-traditional examples. It contains a couple of examples or suggestions that lie outside the average manager’s creative repertoire.

- 3 (Average): The email has some creative elements but relies mostly on common or conventional ideas. While it may offer some new insights, it does not significantly deviate from typical approaches.

- 2 (Below Average): The email lacks creativity, offering only generic or clichéd ideas. It fails to provide new insights or innovative thinking, sticking closely to traditional problem-solving methods.

- 1 (Poor): The email shows no creativity, offering only repetitive, uninspired ideas. It lacks any original thought and fails to provide any new or interesting perspectives on the business issue.

**B3.3. Sample Training Exercise**

Below we provide a business issue prompt and a human-written email in response to address the prompt. This is one of the four emails used in the training sessions. After each evaluator finalized their ratings, we conveyed to them the “correct ratings,” pasted after the email in italic font. We then discussed as a group the rationale for each rating.

*Prompt 1: We are a library located in an urban area. Recently, we have noticed a decreased demand for paper books and an increased demand for electronic resources. As a business manager at our company, write an email to your colleagues about your plan to address this question.*

Dear Colleagues,

Our library has seen a decrease in demand for traditional paper books and an increase in demand for electronic resources. To address this shift, I propose that we expand our digital collection and upgrade our technology infrastructure and offer remote access to our customers.

This plan will require collaboration across departments and attention to current trends. Thank you!

Best regards,

[Your Name] Business Manager

*Ratings:*

*Completeness – 1*

*Relevance – 2*

*Actionability – 1*

*Creativity – 2*

**B3.4. Sample Calibration Exercise**

The calibration was conducted one-on-one with each evaluator via email. We sent them two emails to evaluate, collected their ratings, and engaged in a debrief with comments. Below, we present a sample email exchange in the calibration process, in which the principal investigator calibrated an evaluator’s ratings by telling them the “correct” ratings and explain the rationales.


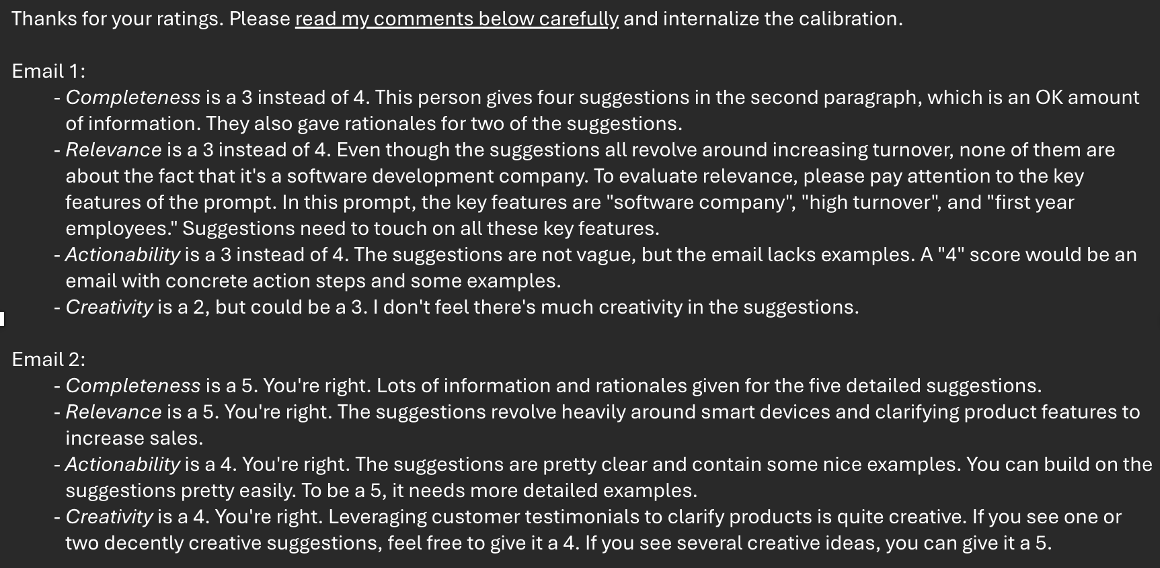


**B3.5 Sample Emails Written by Human Participants**

**English Email**

*Scenario: R&D with AI treatment*

Hey everyone,

I've been thinking a lot about how we can take our sports gear to the next level in terms of safety. Remember how we were talking about that new D3O material at the trade show? It seems like it could be a game-changer for impact absorption while keeping things flexible.

Here's what I propose: Let's do a deep dive into optimizing impact absorption and minimizing injury risk in our gear. We can explore cutting-edge materials like D3O, analyze different foams for specific applications, and even revisit our design principles with a focus on maximum protection.

Think about it - I saw this stat recently about how many injuries could be prevented with a better fit. Maybe we can brainstorm some new fastening systems to ensure a truly snug fit for everyone.

By combining cutting-edge materials with innovative design, we can elevate our protective gear to new levels of safety and performance, ultimately keeping athletes safer on the field.

In the coming days, I'll assign research tasks and create a schedule for brainstorming sessions. Please let me know if you have any initial thoughts or suggestions.

I'm really pumped to dive into this with all of you. Your ideas are invaluable!

Best,

[Name Redacted]

Business Manager

**Arabic Email**

*Scenario: Marketing without AI treatment*

مرحبًا جميعًا،

نظرًا لانخفاض أعداد الزوار في متجرنا المادي، نحن بحاجة إلى تحسين وجودنا الإلكتروني واستراتيجية التسويق الرقمي. الخطوات الأولية تشمل تطوير موقعنا الإلكتروني لجعله أكثر جاذبية وسهولة في التصفح، بالإضافة إلى تحسين استراتيجيات التسويق الرقمي لجذب الجمهور الشاب. أتطلع إلى مشاركة المزيد من التفاصيل والأفكارالمكتوبة ادناه

تطوير الموقع الإلكتروني
سنعمل على إنشاء محتوى متميز ومثير للاهتمام يجذب الجمهور الشاب، بما في ذلك المقالات، والمدونات، والمراجعات الكتابية.
سنركز على تقديم محتوى يتناول القضايا والمواضيع التي تهم الشباب حاليًا، ونضع الكتب ذات الصلة في مقدمة عروضنا.

تحسين محتوى الموقع
سنعمل على إنشاء محتوى متميز ومثير للاهتمام يجذب الجمهور الشاب، بما في ذلك المقالات، والمدونات، والمراجعات الكتابية.
سنركز على تقديم محتوى يتناول القضايا والمواضيع التي تهم الشباب حاليًا، ونضع الكتب ذات الصلة في مقدمة عروضنا .

استخدام التسويق بالمحتوى
سنعتمد استراتيجية تسويق بالمحتوى لجذب الجمهور وبناء علاقات دائمة معهم، عبر إنشاء محتوى قيم ومفيد يثير اهتمامهم.

التعاون مع المؤثرين الرقميين
سنقوم بالتعاون مع مؤثرين رقميين في مجال الكتب والثقافة للمساعدة في الترويج لمتجرنا وكتبنا.

شكرًا لتفهمكم ودعمكم المستمر،

**Chinese Email**

*Scenario: Customer Service without AI treatment*

主題：減少新型高端智能電視退款率之商業計劃

致各位同事：

近期我們留意到我司的新型高端智能電視退款率異常地高，引起了我司的關注。經過調查及查閱顧客退款時的意見回饋，發現大部分顧客都是因為設置複雜所以他們才選擇退款的。有見及此，我制定了以下商業計劃，希望改善問題：


1. 重新設計產品設置介面，簡化設置步驟。
2. 提供示範設置短片供客戶參考。
3. 加強職員培訓，令職員們可以更了解產品特性及設置程序，有助幫助客戶。

希望實施以上措施後，能夠顯著地降低新型高端智能電視退款率，提升客戶滿意度及我司聲譽。如有其他建議，歡迎提出，希望大家能多多支持，謝謝！

業務經理 謹啟

**B4. AI Content Evaluation Materials**

**B4.1. AI Content Evaluators’ Backgrounds**

|  | *Evaluator* | *Gender* | *Program* | *Work Experience* | *Industry* | *Country of Origin* |
| --- | --- | --- | --- | --- | --- | --- |
| English | Evaluator 1 | F | Int’l Studies | 6 years | Investment  Law | U.S. |
|  | Evaluator 2 | M | Engineering | 1 year | Healthcare | India |
| Arabic | Evaluator 3 | F | Public Management | 5 years | Consulting,  Government | Saudi Arabia |
|  | Evaluator 4 | F | Communications | 5 years | Consulting | Egypt |
| Chinese | Evaluator 5 | M | Economics | 2 years | Finance,  Energy | China |
|  | Evaluator 6 | F | Economics | 2 years | Energy,  Investment | China |

**B4.2. AI Content Evaluation Criteria**

**Completeness:**

- 5 (Excellent): The response thoroughly addresses all aspects of the prompt, providing a comprehensive and detailed answer. It offers rich information on multiple aspects of the prompt and explains the rationales for its suggestions.
- 4 (Good): The response covers most aspects of the prompt in detail, and it gives some explanations for its recommendations. It provides a nearly complete answer, though there may be minor areas that could be further enriched and elaborated upon.
- 3 (Average): The response addresses the main points of the prompt but lacks depth, information, and examples in certain areas. Some aspects are not fully explored, leaving room for additional information or clarification.
- 2 (Below Average): The response addresses only a few elements of the prompt, leaving significant gaps in the information provided. It lacks depth and breadth, failing to cover important aspects of the topic or give good examples. It gives little explanation for its recommendations.
- 1 (Poor): The response barely addresses the prompt, missing key components and providing a very superficial overview. It leaves most of the query unanswered and fails to provide a useful understanding of the topic. It gives no explanation for its recommendations.

**Relevance:**

- 5 (Excellent): The response directly addresses the prompt with highly focused and pertinent information. It stays on topic throughout and all information provided is clearly related to the query, enhancing the user’s understanding of the subject.
- 4 (Good): The response is mostly focused on the prompt, with most information being relevant and topical. There may be minor digressions, but they do not significantly detract from the relevance of the answer.
- 3 (Average): The response addresses the prompt but includes some irrelevant or tangential information. While the core of the response is relevant, there are noticeable deviations from the main topic.
- 2 (Below Average): The response only partially addresses the prompt, with much of the information being irrelevant or off-topic. It frequently deviates from the query and fails to stay focused on the main subject.
- 1 (Poor): The response largely fails to address the prompt, consisting mainly of irrelevant or unrelated information. It does not provide a meaningful or relevant answer to the query.

**Actionability:**

- 5 (Excellent): The response provides highly actionable and practical advice or solutions. It offers clear guidance or specific strategies that can be easily implemented. It gives rich examples that illustrate how its recommendations can be implemented. It also opens the door to further learning through additional questions and follow-up interactions. It also has a high level of cultural appropriateness.
- 4 (Good): The response is actionable, with practical advice, solutions, and examples. It provides clear guidance or strategies that are generally easy to implement, though there may be minor areas lacking in practicality and possibility for further engagement and learning. It is also culturally appropriate.
- 3 (Average): The response offers some actionable advice, but it may be somewhat vague or lacking in illustrations. While it provides a direction or solution, more specific details or steps may be needed for effective implementation and for further interaction to learn more about the topic. It might or might not be culturally appropriate.
- 2 (Below Average): The response offers limited actionable advice, with solutions or suggestions that are difficult to implement and follow up. It lacks practicality and illustrations, and it fails to provide clear or detailed guidance. It might lack in cultural appropriateness.
- 1 (Poor): The response provides no actionable advice or practical solutions. It offers no clear guidance or examples, leaving the query unresolved and failing to address practical implementation and follow-up learning. It might contain suggestions that are highly inappropriate given the cultural context.

**Creativity:**

- 5 (Excellent): The response demonstrates exceptional creativity, offering unique, innovative ideas or solutions. It shows original thinking and presents multiple concepts or examples that are novel with respect to the average manager’s creative repertoire.
- 4 (Good): The response is creative, providing fresh insights or approaches that are not usually thought of by the average manager. It goes beyond standard ideas, offering new ways of thinking about the topic and non-traditional examples.
- 3 (Average): The response has some creative elements but relies mostly on common or conventional ideas. While it may offer some new insights, it does not significantly deviate from typical thinking.
- 2 (Below Average): The response lacks creativity, offering only generic or clichéd ideas. It fails to provide new insights or innovative thinking, sticking closely to traditional managerial thinking.
- 1 (Poor): The response shows no creativity, offering only repetitive, uninspired ideas. It lacks any original thought and fails to provide any new or interesting perspectives on the topic.

1. **Supplementary Figures**

**Fig. S1. Experimental Design in Study 2**

**Arabic**

160 participants

**Chinese**

160 participants

**English**

160 participants

**Onboarding**

Same for each language: recruitment message, linking to Qualtrics, informed consent

**Email-Writing Task**

16 scenarios

| *Control*  (8 out of 16 scenarios)  Asked to write email to prompt  without AI assistance | *AI Treatment*  (8 out of 16 scenarios)  Asked to write email, providing them with an AI-generated response to prompt |
| --- | --- |

**Post-Task Questions**

AI usage, confidence, difficulty, feeling about task

**End of Survey**

**Language Screening**

3 concurrent projects on survey platform (English, Arabic, Chinese)

**Fig. S2. AI Content Evaluation Boxplots**


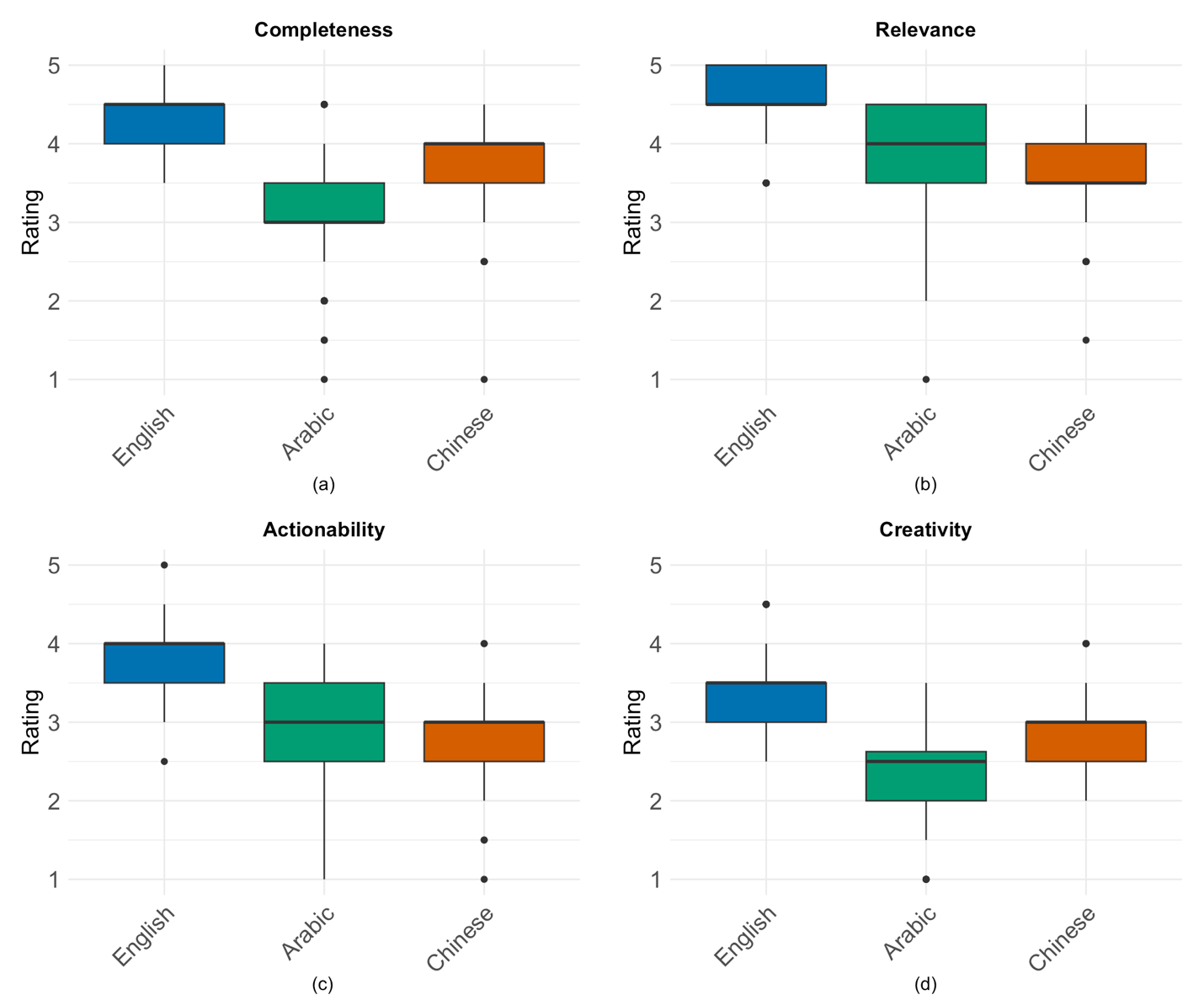


**Fig. S3. AI Content Evaluation By Work Type**


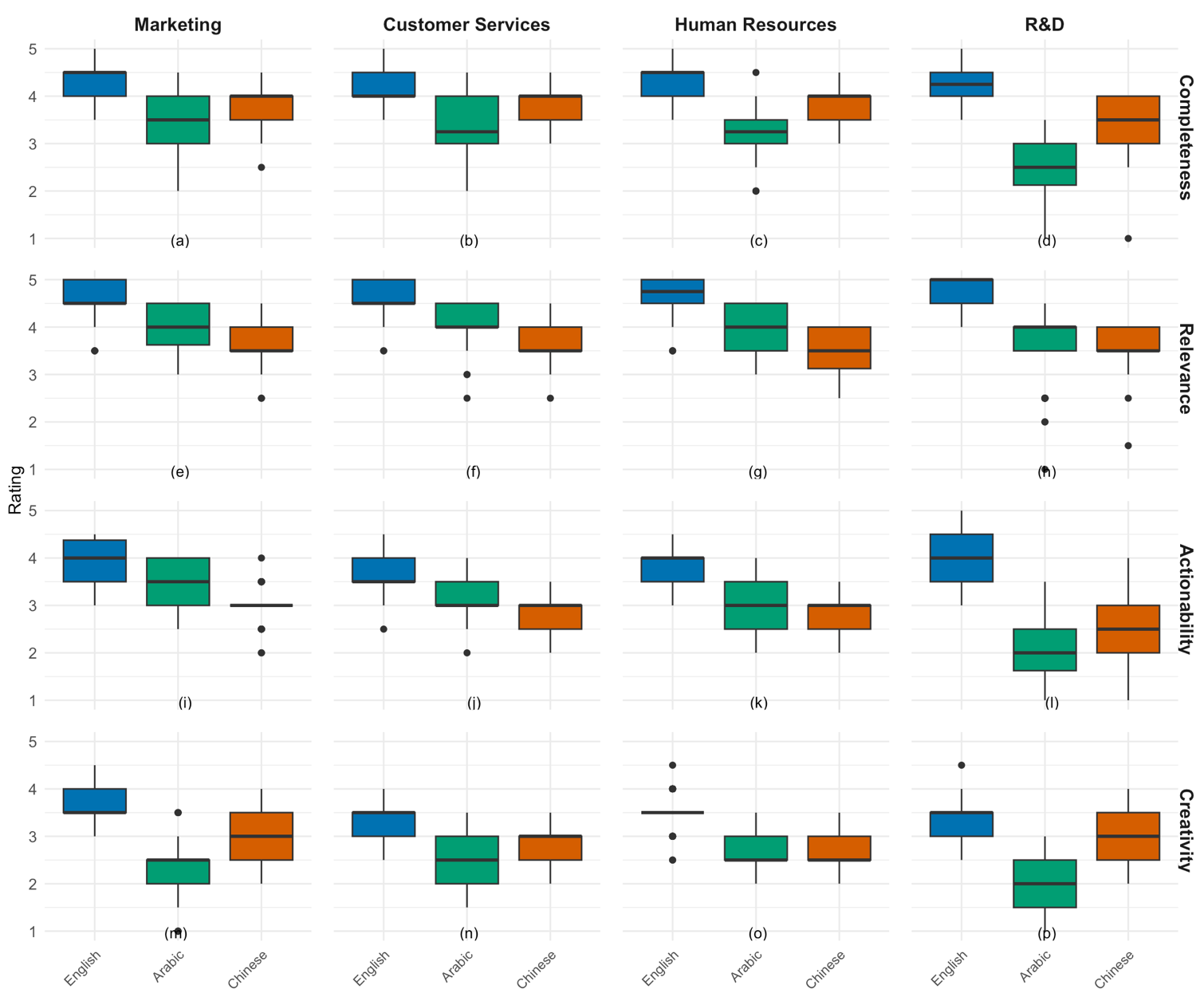


**Fig. S4. Email Length by Language and AI Treatment**


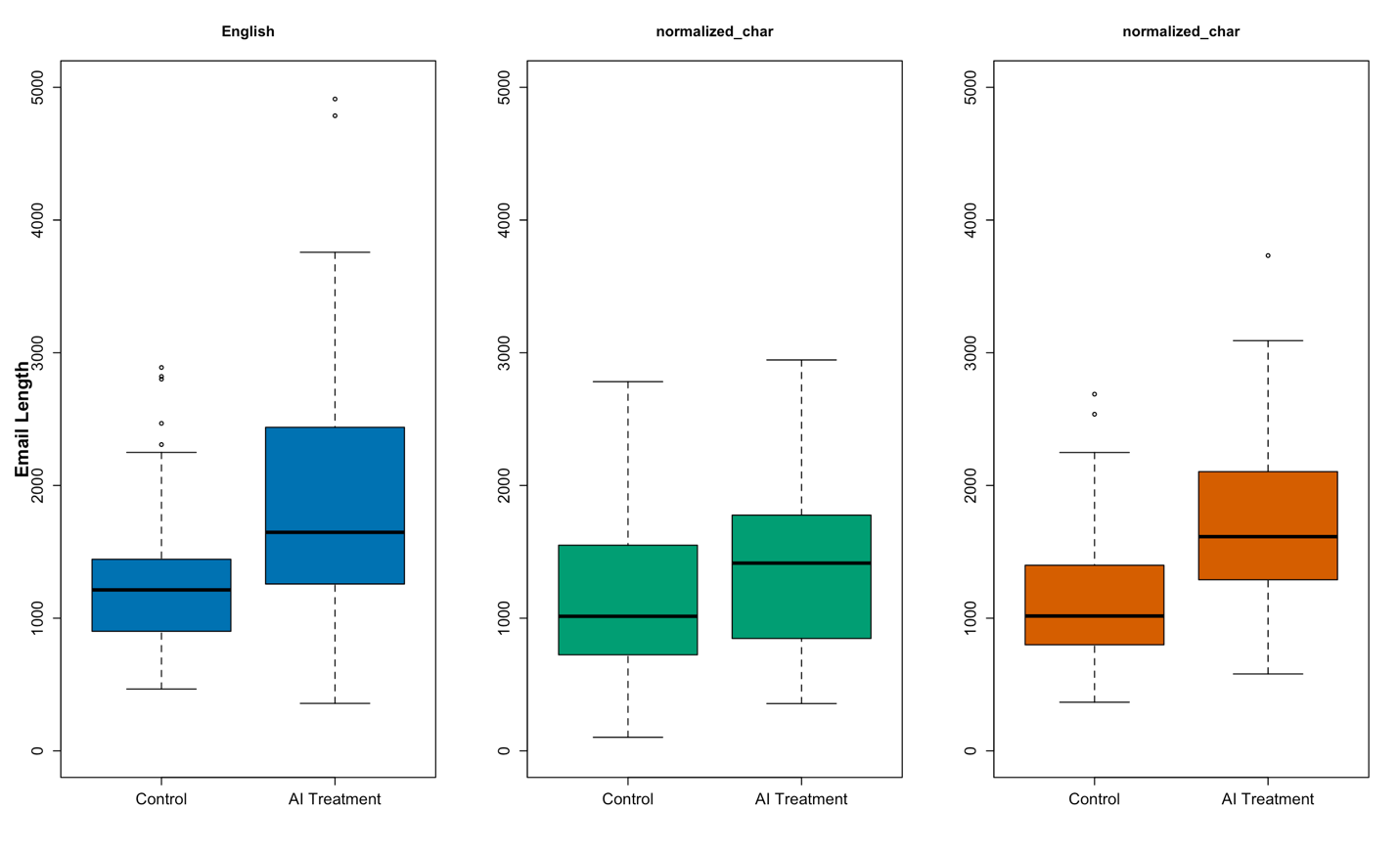


**Fig. S5. Time Taken to Write Emails by Language and AI Treatment**


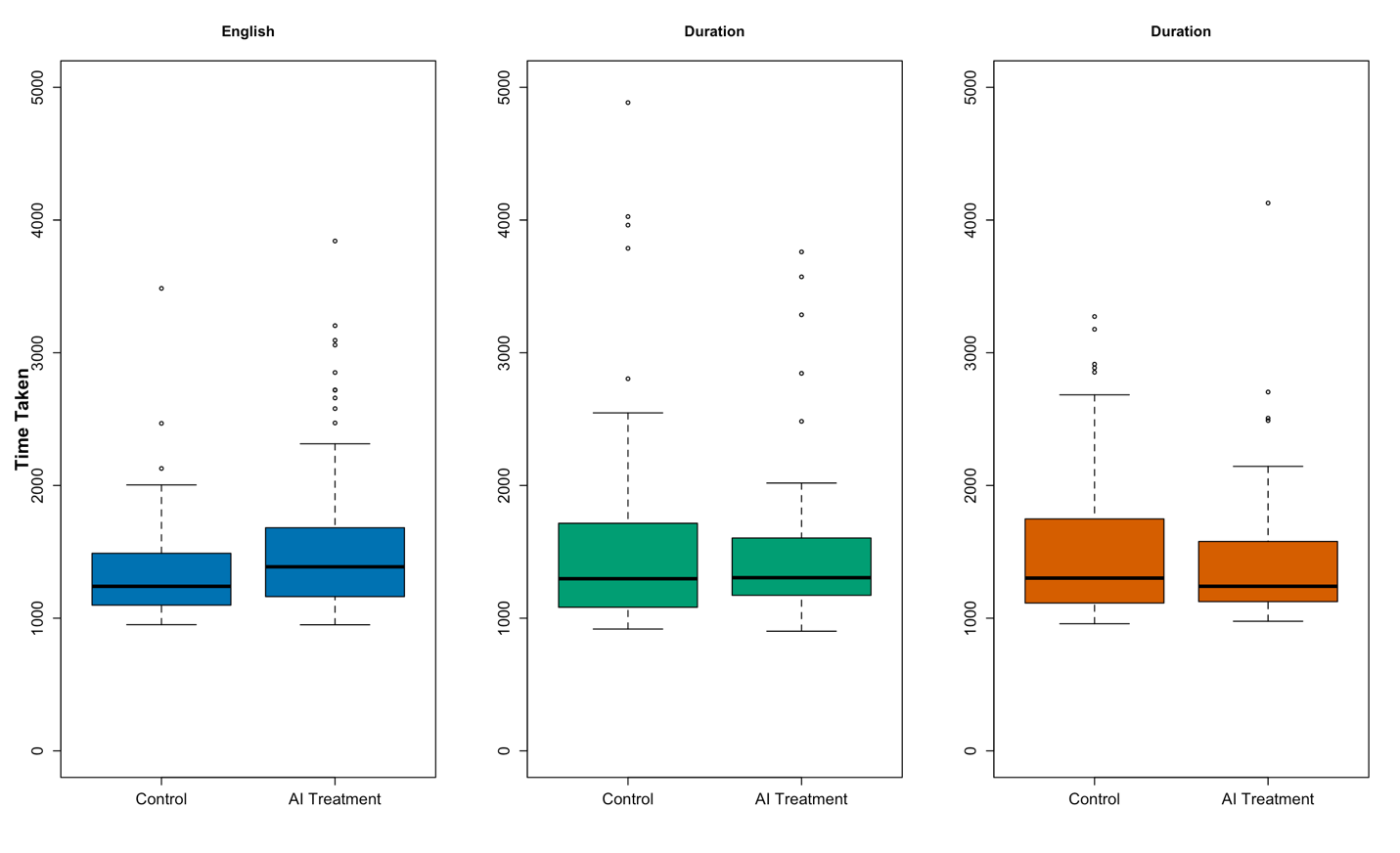


**Fig. S6. Self-Reported Confidence About Email Task by AI Treatment**


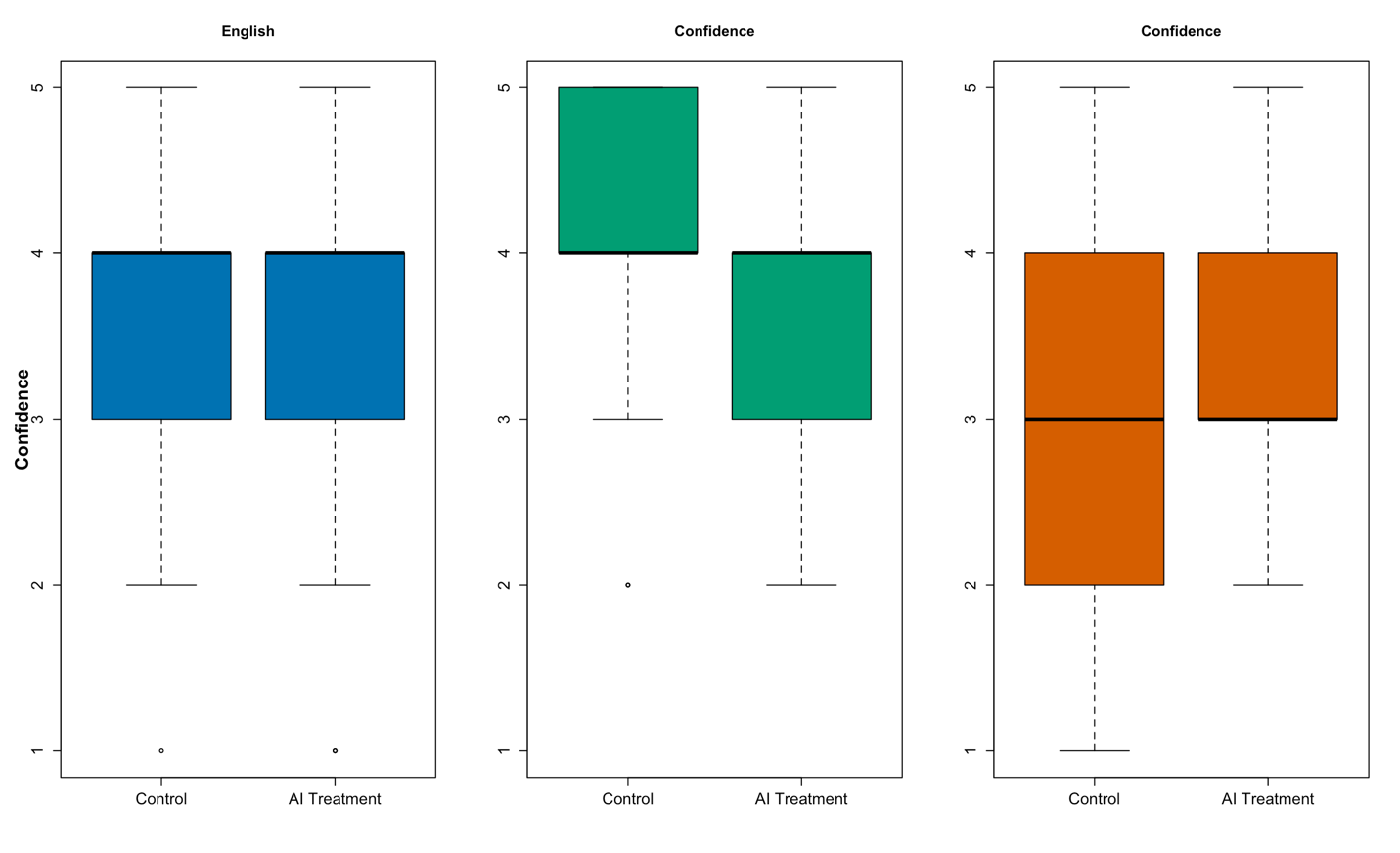


**Fig. S7. Self-Reported Difficulty of Email Task by AI Treatment**


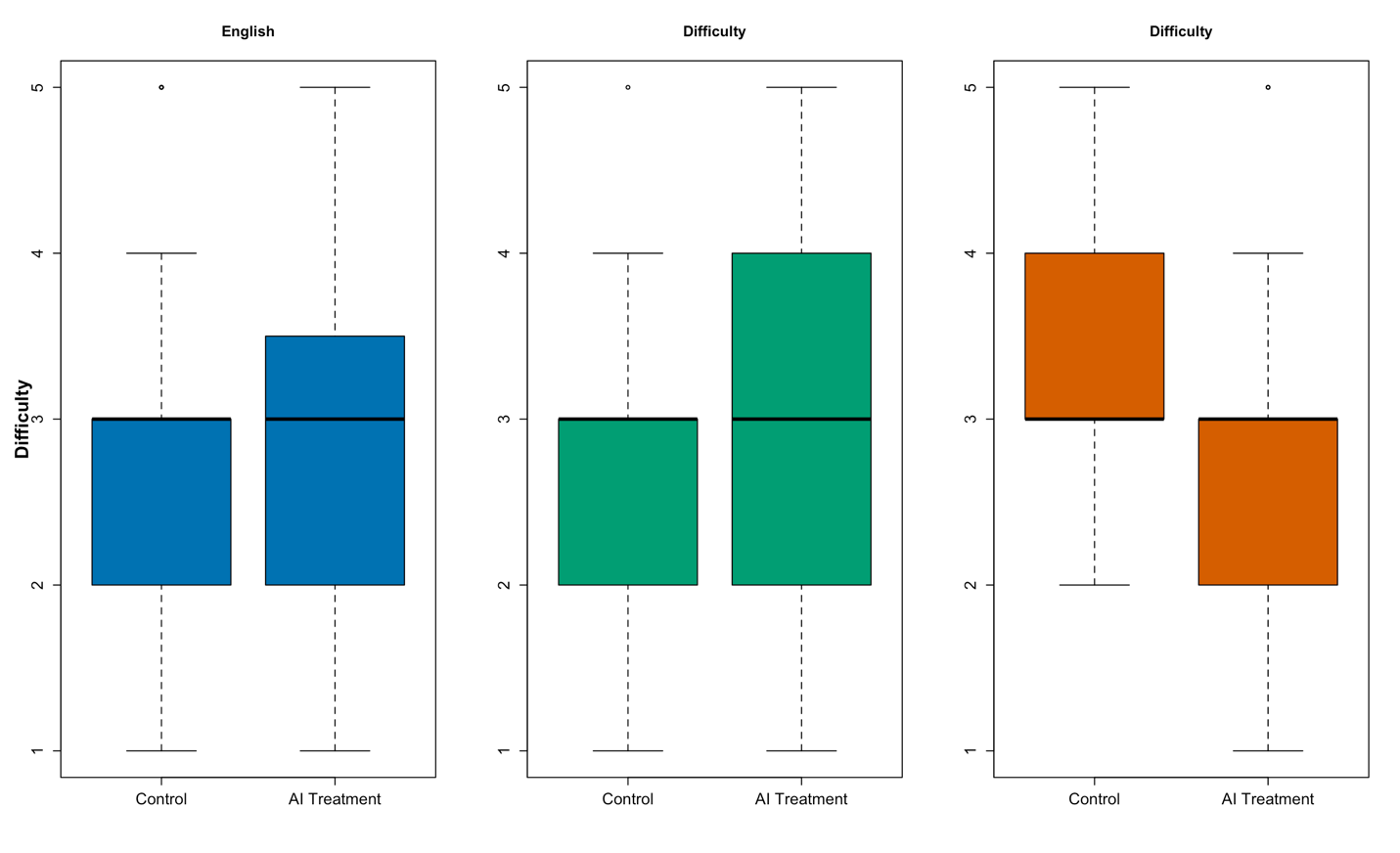


1. **Supplementary Tables**

**Table S1. Evaluation of the Quality of AI-Generated Content**

|  | *Completeness* | *Relevance* | *Actionability* | *Creativity* |
| --- | --- | --- | --- | --- |
|  | (1) | (2) | (3) | (4) |
|  | | | | |
| Arabic | -1.133^***^ | -0.750^***^ | -0.893^***^ | -1.105^***^ |
|  | (0.050) | (0.048) | (0.055) | (0.046) |
| Chinese | -0.592^***^ | -1.090^***^ | -1.077^***^ | -0.600^***^ |
|  | (0.050) | (0.048) | (0.055) | (0.046) |
| Constant | 4.242^***^ | 4.613^***^ | 3.823^***^ | 3.068^***^ |
|  | (0.291) | (0.277) | (0.317) | (0.266) |
|  | | | | |
| Observations | 600 | 600 | 600 | 600 |
| R^2^ | 0.659 | 0.653 | 0.633 | 0.686 |
| F Statistic (df = 201; 398) | 3.822^***^ | 3.732^***^ | 3.412^***^ | 4.328^***^ |

*Note:* This table reports language treatment effects on the quality of AI content generated with ChatGPT-4. Section 1.5 in the supplementary information presents more detail on the four quality dimensions. AI content is in the form of AI-generated response to address a hypothetical business issue. For each AI response, trained evaluators gave ratings of 1 to 5 along each dimension. The baseline group (represented by the Constant coefficients) in the regressions is “English”. Standard errors clustered at the participant-level in parentheses. *p<0.1; **p<0.05; ***p<0.01.

**Table S2. AI Content Evaluation Across Work Task Types**

|  | | | | |
| --- | --- | --- | --- | --- |
|  | *Completeness* | *Relevance* | *Actionability* | *Creativity* |
|  | (1) | (2) | (3) | (4) |
| Arabic | -1.050^***^ | -0.570^***^ | -0.580^***^ | -1.230^***^ |
|  | (0.096) | (0.092) | (0.096) | (0.087) |
| Chinese | -0.680^***^ | -1.020^***^ | -0.970^***^ | -0.680^***^ |
|  | (0.096) | (0.092) | (0.096) | (0.087) |
| Customer Services | -0.183 | 0.183 | -0.090 | -0.820^**^ |
|  | (0.401) | (0.385) | (0.400) | (0.362) |
| Human Resources | 0.130 | 0.577 | 0.577 | -0.287 |
|  | (0.401) | (0.385) | (0.400) | (0.362) |
| R&D | 0.380 | 0.740^*^ | 0.407 | -0.167 |
|  | (0.401) | (0.385) | (0.400) | (0.362) |
| Arabic × Customer Service | 0.280^**^ | -0.030 | 0.130 | 0.310^**^ |
|  | (0.136) | (0.131) | (0.136) | (0.123) |
| Chinese × Customer Service | 0.270^**^ | -0.020 | 0.140 | 0.150 |
|  | (0.136) | (0.131) | (0.136) | (0.123) |
| Arabic × Human Resources | -0.090 | -0.090 | -0.170 | 0.380^***^ |
|  | (0.136) | (0.131) | (0.136) | (0.123) |
| Chinese × Human Resources | 0.200 | -0.140 | -0.060 | -0.020 |
|  | (0.136) | (0.131) | (0.136) | (0.123) |
| Arabic × R&D | -0.520^***^ | -0.600^***^ | -1.210^***^ | -0.190 |
|  | (0.136) | (0.131) | (0.136) | (0.123) |
| Chinese × R&D | -0.120 | -0.120 | -0.510^***^ | 0.190 |
|  | (0.136) | (0.131) | (0.136) | (0.123) |
| Constant | 4.243^***^ | 4.363^***^ | 3.683^***^ | 3.803^***^ |
|  | (0.283) | (0.272) | (0.283) | (0.256) |
|  | | | | |
| Observations | 600 | 600 | 600 | 600 |
| R^2^ | 0.690 | 0.681 | 0.720 | 0.722 |
| F Statistic (df = 207; 392) | 4.221^***^ | 4.047^***^ | 4.870^***^ | 4.914^***^ |

***Note:*** This table reports the evaluation of the quality of AI-generated content, with task type as a an interaction term. The baseline language group is “English,” and the baseline task type is “Marketing.” Standard errors clustered at the participant-level in parentheses. *p<0.1; **p<0.05; ***p<0.01.

| **Table S3. Descriptive Statistics and Balance Tests Across AI Treatment**   \|  \| Control (N=235) \| \| Treatment (N=224) \| \|  \| \| --- \| --- \| --- \| --- \| --- \| --- \| \|  \| Mean \| S.D. \| Mean \| S.D. \| Difference \| \| Age \| 30.78 \| 9.13 \| 30.32 \| 9.05 \| -0.46 [0.60] \| \| Female \| 0.50 \| 0.50 \| 0.46 \| 0.50 \| -0.04 [0.45] \| \| White \| 0.33 \| 0.47 \| 0.31 \| 0.46 \| -0.02 [0.62] \| \| Asian \| 0.33 \| 0.47 \| 0.35 \| 0.48 \| 0.02 [0.73] \| \| Black \| 0.12 \| 0.33 \| 0.13 \| 0.34 \| 0.01 [0.81] \| \| Student \| 0.32 \| 0.47 \| 0.40 \| 0.49 \| 0.09 [0.06] \| \| Full Time \| 0.44 \| 0.50 \| 0.40 \| 0.49 \| -0.04 [0.46] \| \| Second Language \| 0.47 \| 0.50 \| 0.45 \| 0.50 \| -0.02 [0.70] \|   *Note:* This table presents the summary statistics of demographic information in control versus treatment groups. In the Difference column, p-values are included in brackets.  **Table S4. Descriptive Statistics Across Language Groups**   \|  \| English (N=153) \| \| Arabic (N=123) \| \| Chinese (N=147) \| \| \| --- \| --- \| --- \| --- \| --- \| --- \| --- \| \|  \| Mean \| S.D. \| Mean \| S.D. \| Difference \| S.D. \| \| Age \| 28.61 \| 7.95 \| 29.86 \| 8.94 \| 33.71 \| 9.71 \| \| Female \| 0.49 \| 0.50 \| 0.36 \| 0.48 \| 0.56 \| 0.50 \| \| White \| 0.58 \| 0.49 \| 0.34 \| 0.48 \| 0.04 \| 0.20 \| \| Asian \| 0.03 \| 0.16 \| 0.06 \| 0.24 \| 0.89 \| 0.32 \| \| Black \| 0.28 \| 0.45 \| 0.07 \| 0.26 \| 0.01 \| 0.12 \| \| Student \| 0.38 \| 0.49 \| 0.45 \| 0.50 \| 0.27 \| 0.44 \| \| Full Time \| 0.37 \| 0.48 \| 0.45 \| 0.50 \| 0.46 \| 0.50 \| \| Second Language \| 0.63 \| 0.49 \| 0.41 \| 0.49 \| 0.33 \| 0.47 \|   *Note:* This table presents the descriptive statistics of demographic information in the three language groups: English, Arabic, and Chinese.  **Table S5. Effects of Language on Email Quality**   \|  \| *Completeness* \| *Relevance* \| *Actionability* \| *Creativity* \| \| --- \| --- \| --- \| --- \| --- \| \|  \| (1) \| (2) \| (3) \| (4) \| \|  \| \| \| \| \| \| AI Treatment \| 0.550^***^ \| 0.512^***^ \| 0.889^***^ \| 0.998^***^ \| \|  \| (0.146) \| (0.142) \| (0.144) \| (0.159) \| \| Arabic \| -0.176 \| 0.087 \| -0.110 \| -0.284^*^ \| \|  \| (0.177) \| (0.168) \| (0.171) \| (0.169) \| \| Chinese \| -0.145 \| 0.158 \| 0.048 \| -0.044 \| \|  \| (0.150) \| (0.161) \| (0.153) \| (0.151) \| \| AI Treatment × Arabic \| 0.119 \| 0.078 \| -0.572^**^ \| -0.357 \| \|  \| (0.248) \| (0.220) \| (0.233) \| (0.240) \| \| AI Treatment × Chinese \| 0.193 \| 0.068 \| -0.461^**^ \| -0.499^**^ \| \|  \| (0.213) \| (0.224) \| (0.200) \| (0.206) \| \| Constant \| 3.305^***^ \| 3.429^***^ \| 3.065^***^ \| 2.844^***^ \| \|  \| (0.105) \| (0.102) \| (0.106) \| (0.112) \|   ***Note:* This table presents the effects of AI treatment on the quality of the emails written by human participants across languages. The four dimensions of quality the evaluators used to rate the mails are: Completeness, Relevance, Actionability, and Creativity.** Standard errors clustered at the participant-level in parentheses. *p<0.1; **p<0.05; ***p<0.01.  **Table S6. Interrater Reliability Within Language Groups**   \|  \| *Evaluator* \| *Completeness* \| *Relevance* \| *Actionability* \| *Creativity* \| \| --- \| --- \| --- \| --- \| --- \| --- \| \| English \| Evaluator 1 \| 0.71  [0.000] \| 0.71  [0.000] \| 0.82  [0.000] \| 0.83  [0.000] \| \| Evaluator 2 \| \| Arabic \| Evaluator 3 \| 0.88  [0.000] \| 0.77  [0.000] \| 0.75  [0.000] \| 0.72  [0.000] \| \| Evaluator 4 \| \| Chinese \| Evaluator 5 \| 0.87  [0.000] \| 0.78  [0.000] \| 0.85  [0.000] \| 0.73  [0.000] \| \| Evaluator 6 \|   *Note:* This table reports the Kendall’s *W* coefficients between pairs of email evaluators for each language group. The p-values reported in brackets indicate the likelihood of rejecting the null of “the evaluations show no agreement.”  **Table S7. Prompt Length as Interaction Term**   \|  \| \| \| \| \| \| --- \| --- \| --- \| --- \| --- \| \|  \| *Completeness* \| *Relevance* \| *Actionability* \| *Creativity* \| \|  \| (1) \| (2) \| (3) \| (4) \| \| AI Treatment \| -0.527 \| -1.425 \| -1.188 \| -1.167 \| \|  \| (1.463) \| (1.437) \| (1.414) \| (1.575) \| \| Arabic \| -2.065 \| -2.664 \| -3.613^**^ \| -2.249 \| \|  \| (1.785) \| (2.006) \| (1.824) \| (1.697) \| \| Chinese \| -0.189 \| -0.962 \| -0.361 \| -0.261 \| \|  \| (1.291) \| (1.410) \| (1.411) \| (1.332) \| \| Prompt Length \| -0.003 \| -0.006^**^ \| -0.005 \| -0.003 \| \|  \| (0.003) \| (0.003) \| (0.003) \| (0.003) \| \| AI Treatment × Arabic \| 1.298 \| 1.901 \| 1.865 \| 3.002 \| \|  \| (2.498) \| (2.492) \| (2.391) \| (2.456) \| \| AI Treatment × Chinese \| 2.705 \| 2.584 \| 1.044 \| 0.084 \| \|  \| (2.171) \| (2.326) \| (1.995) \| (2.044) \| \| AI Treatment × Prompt Length \| 0.003 \| 0.006^**^ \| 0.005^*^ \| 0.004 \| \|  \| (0.003) \| (0.003) \| (0.003) \| (0.003) \| \| Arabic × Prompt Length \| 0.006 \| 0.008 \| 0.010^**^ \| 0.006 \| \|  \| (0.005) \| (0.006) \| (0.005) \| (0.005) \| \| Chinese × Prompt Length \| 0.0004 \| 0.004 \| 0.002 \| 0.001 \| \|  \| (0.004) \| (0.004) \| (0.004) \| (0.004) \| \| AI Treatment × Arabic:Prompt Length \| -0.005 \| -0.008 \| -0.009^*^ \| -0.006 \| \|  \| (0.005) \| (0.006) \| (0.005) \| (0.005) \| \| AI Treatment × Chinese:Prompt Length \| -0.002 \| -0.004 \| -0.002 \| -0.001 \| \|  \| (0.004) \| (0.004) \| (0.004) \| (0.004) \| \| Constant \| 4.313^***^ \| 5.353^***^ \| 4.689^***^ \| 3.900^***^ \| \|  \| (0.936) \| (0.965) \| (0.999) \| (0.963) \|   ***Note:* This table** presents the AI treatment effects across languages, with prompt length as an interaction term. Standard errors clustered at the participant-level in parentheses. *p<0.1; **p<0.05; ***p<0.01.  **Table S8. Long and Short Prompts**   \|  \| *Completeness* \| *Relevance* \| *Actionability* \| *Creativity* \| \| --- \| --- \| --- \| --- \| --- \| \|  \| (1) \| (2) \| (3) \| (4) \| \| AI Treatment \| 0.972^***^ \| 0.674^**^ \| 1.174^***^ \| 1.518^***^ \| \|  \| (0.262) \| (0.288) \| (0.249) \| (0.307) \| \| Arabic \| -0.145 \| -0.042 \| -0.028 \| -0.066 \| \|  \| (0.284) \| (0.289) \| (0.262) \| (0.307) \| \| Chinese \| -0.189 \| 0.017 \| 0.061 \| 0.096 \| \|  \| (0.252) \| (0.298) \| (0.253) \| (0.296) \| \| Longer Prompt (Arabic/Chinese) \| 0.109 \| -0.051 \| 0.121 \| 0.277 \| \|  \| (0.235) \| (0.250) \| (0.230) \| (0.274) \| \| AI Treatment × Arabic \| -0.062 \| 0.083 \| -0.749^**^ \| -0.760^*^ \| \|  \| (0.382) \| (0.375) \| (0.343) \| (0.399) \| \| AI Treatment × Chinese \| -0.213 \| 0.083 \| -0.734^**^ \| -0.875^**^ \| \|  \| (0.352) \| (0.393) \| (0.329) \| (0.376) \| \| AI Treatment × Longer Prompt (Arabic/Chinese) \| -0.572^*^ \| -0.229 \| -0.382 \| -0.692^*^ \| \|  \| (0.313) \| (0.332) \| (0.303) \| (0.359) \| \| Arabic × Longer Prompt (Arabic/Chinese) \| 0.005 \| 0.234 \| -0.094 \| -0.273 \| \|  \| (0.371) \| (0.367) \| (0.355) \| (0.374) \| \| Chinese × Longer Prompt (Arabic/Chinese) \| 0.130 \| 0.236 \| 0.032 \| -0.131 \| \|  \| (0.318) \| (0.357) \| (0.320) \| (0.345) \| \| AI Treatment × Arabic:Longer Prompt (Arabic/Chinese) \| 0.068 \| -0.119 \| 0.155 \| 0.446 \| \|  \| (0.508) \| (0.473) \| (0.479) \| (0.508) \| \| AI Treatment × Chinese:Longer Prompt (Arabic/Chinese) \| 0.546 \| -0.097 \| 0.362 \| 0.427 \| \|  \| (0.443) \| (0.483) \| (0.414) \| (0.448) \| \| Constant \| 3.219^***^ \| 3.469^***^ \| 2.969^***^ \| 2.625^***^ \| \|  \| (0.200) \| (0.222) \| (0.193) \| (0.244) \|   ***Note:* This table** presents the AI treatment effects across languages, with the binary variable Longer Prompt (Arabic/Chinese) as an interaction term. That variable equals 1 if the ratio of the length of an Arabic or Chinese prompt to that of the corresponding English prompt is greater than the sample median, and 0 otherwise. Standard errors clustered at the participant-level in parentheses. *p<0.1; **p<0.05; ***p<0.01.  **Table S9. Effects of Language on Email Length** | | | | |
| --- | --- | --- | --- | --- | --- | --- | --- | --- | --- | --- | --- | --- | --- | --- | --- | --- | --- | --- | --- | --- | --- | --- | --- | --- | --- | --- | --- | --- | --- | --- | --- | --- | --- | --- | --- | --- | --- | --- | --- | --- | --- | --- | --- | --- | --- | --- | --- | --- | --- | --- | --- | --- | --- | --- | --- | --- | --- | --- | --- | --- | --- | --- | --- | --- | --- | --- | --- | --- | --- | --- | --- | --- | --- | --- | --- | --- | --- | --- | --- | --- | --- | --- | --- | --- | --- | --- | --- | --- | --- | --- | --- | --- | --- | --- | --- | --- | --- | --- | --- | --- | --- | --- | --- | --- | --- | --- | --- | --- | --- | --- | --- | --- | --- | --- | --- | --- | --- | --- | --- | --- | --- | --- | --- | --- | --- | --- | --- | --- | --- | --- | --- | --- | --- | --- | --- | --- | --- | --- | --- | --- | --- | --- | --- | --- | --- | --- | --- | --- | --- | --- | --- | --- | --- | --- | --- | --- | --- | --- | --- | --- | --- | --- | --- | --- | --- | --- | --- | --- | --- | --- | --- | --- | --- | --- | --- | --- | --- | --- | --- | --- | --- | --- | --- | --- | --- | --- | --- | --- | --- | --- | --- | --- | --- | --- | --- | --- | --- | --- | --- | --- | --- | --- | --- | --- | --- | --- | --- | --- | --- | --- | --- | --- | --- | --- | --- | --- | --- | --- | --- | --- | --- | --- | --- | --- | --- | --- | --- | --- | --- | --- | --- | --- | --- | --- | --- | --- | --- | --- | --- | --- | --- | --- | --- | --- | --- | --- | --- | --- | --- | --- | --- | --- | --- | --- | --- | --- | --- | --- | --- | --- | --- | --- | --- | --- | --- | --- | --- | --- | --- | --- | --- | --- | --- | --- | --- | --- | --- | --- | --- | --- | --- | --- | --- | --- | --- | --- | --- | --- | --- | --- | --- | --- | --- | --- | --- | --- | --- | --- | --- | --- | --- | --- | --- | --- | --- | --- | --- | --- | --- | --- | --- | --- | --- | --- | --- | --- | --- | --- | --- | --- | --- | --- | --- | --- | --- | --- | --- | --- | --- | --- | --- | --- | --- | --- | --- | --- | --- | --- | --- | --- | --- | --- | --- | --- | --- | --- | --- | --- | --- | --- | --- | --- | --- | --- | --- | --- | --- | --- | --- | --- | --- | --- | --- | --- | --- | --- | --- | --- | --- | --- | --- | --- | --- | --- | --- | --- | --- | --- | --- | --- | --- | --- | --- | --- | --- | --- | --- | --- | --- | --- | --- | --- | --- | --- | --- | --- | --- | --- | --- | --- | --- | --- | --- | --- | --- | --- | --- | --- | --- | --- | --- | --- | --- | --- | --- | --- | --- | --- | --- | --- | --- | --- | --- | --- | --- | --- | --- | --- | --- | --- | --- | --- | --- | --- | --- | --- | --- | --- | --- | --- | --- | --- | --- | --- | --- | --- | --- | --- | --- | --- | --- | --- | --- | --- | --- | --- | --- | --- | --- | --- | --- | --- | --- | --- | --- | --- | --- | --- | --- | --- | --- | --- | --- | --- | --- | --- | --- | --- | --- | --- | --- | --- | --- | --- | --- | --- | --- | --- | --- | --- | --- | --- | --- | --- | --- | --- | --- | --- | --- | --- | --- |
|  | | | | |
|  | DV: Email Length | | | |
|  |  | | | |
|  |  | | | |
|  | (1)  Full Sample | (2)  English | (3)  Arabic | (4)  Chinese |
|  | | | | |
| AI Treatment | 631.386^***^ | 631.386^***^ | 283.382^***^ | 531.799^***^ |
|  | (123.242) | (123.172) | (106.857) | (93.539) |
| Arabic | -156.704^*^ |  |  |  |
|  | (92.472) |  |  |  |
| Chinese | -122.220 |  |  |  |
|  | (84.124) |  |  |  |
| AI Treatment × Arabic | -348.004^**^ |  |  |  |
|  | (163.043) |  |  |  |
| AI Treatment × Chinese | -99.587 |  |  |  |
|  | (154.736) |  |  |  |
| Constant | 1,280.338^***^ | 1,280.338^***^ | 1,123.633^***^ | 1,158.117^***^ |
|  | (59.035) | (59.002) | (71.251) | (59.914) |

*Note:* This table presents the AI treatment effects across languages on email length. Email length is calculated as normalized character count. For English emails, the normalized character count equals the original character count. For Arabic emails, it equals the original character count multiplied by 1.1. For Chinese emails, it equals the original character count multiplied by 2.8. The multipliers are determined by average differences in character count between the three languages and derived from comparing the character counts between professionally translated documents and the English originals in this study. Standard errors clustered at the participant-level in parentheses. *p<0.1; **p<0.05; ***p<0.01.

| **Table S10. Effects of Language on Time Taken to Write Email** | | | | |
| --- | --- | --- | --- | --- |
|  | *DV: Time Taken* | | | |
|  |  | | | |
|  | (1) | (2) | (3) | (4) |
|  | | | | |
| AI Treatment | 233.779^***^ | 233.779^***^ | -137.142 | -92.766 |
|  | (83.872) | (83.824) | (145.936) | (87.598) |
| Arabic | 302.030^**^ |  |  |  |
|  | (130.363) |  |  |  |
| Chinese | 160.098^**^ |  |  |  |
|  | (79.827) |  |  |  |
| AI Treatment × Arabic | -370.921^**^ |  |  |  |
|  | (168.187) |  |  |  |
| AI Treatment × Chinese | -326.545^***^ |  |  |  |
|  | (121.295) |  |  |  |
| Constant | 1,355.182^***^ | 1,355.182^***^ | 1,657.212^***^ | 1,515.280^***^ |
|  | (45.694) | (45.669) | (122.221) | (65.436) |

*Note:* This table presents the AI treatment effects across languages on the time it took for a participant to write the email. Time Taken is measured in seconds. Standard errors clustered at the participant-level in parentheses. *p<0.1; **p<0.05; ***p<0.01.

**Table S11. Effects of Language on Self-Reported Confidence About the Email Task**

|  | *DV: Confidence* | | | |
| --- | --- | --- | --- | --- |
|  |  | | | |
|  |  | | | |
|  | (1) | (2) | (3) | (4) |
|  | | | | |
| AI TreatmentAI Treatment | -0.006 | -0.006 | -0.301^**^ | 0.336^**^ |
|  | (0.153) | (0.153) | (0.137) | (0.141) |
| Arabic | 0.558^***^ |  |  |  |
|  | (0.134) |  |  |  |
| Chinese | -0.466^***^ |  |  |  |
|  | (0.145) |  |  |  |
| Treatment × Arabic | -0.295 |  |  |  |
|  | (0.205) |  |  |  |
| AI Treatment × Chinese | 0.342^*^ |  |  |  |
|  | (0.208) |  |  |  |
| Constant | 3.532^***^ | 3.532^***^ | 4.091^***^ | 3.067^***^ |
|  | (0.101) | (0.101) | (0.088) | (0.105) |

*Note:* This table presents the AI treatment effects across languages on participants’ self-reported confidence about the quality of their email output. Confidence is operationalized on a 1-5 Likert scale (1 = very unconfident; 5= very confident). Standard errors clustered at the participant-level in parentheses. *p<0.1; **p<0.05; ***p<0.01.

**Table S12. Effects of Language on Self-Reported Difficulty of the Email Task**

|  | *DV: Difficulty* | | | |
| --- | --- | --- | --- | --- |
|  |  | | | |
|  |  | | | |
|  | (1) | (2) | (3) | (4) |
|  | | | | |
| AI Treatment | -0.013 | -0.013 | 0.164 | -0.419^***^ |
|  | (0.140) | (0.140) | (0.162) | (0.141) |
| Arabic | -0.156 |  |  |  |
|  | (0.146) |  |  |  |
| Chinese | 0.306^**^ |  |  |  |
|  | (0.134) |  |  |  |
| AI Treatment × Arabic | 0.178 |  |  |  |
|  | (0.214) |  |  |  |
| AI Treatment × Chinese | -0.405^**^ |  |  |  |
|  | (0.199) |  |  |  |
| Constant | 2.974^***^ | 2.974^***^ | 2.818^***^ | 3.280^***^ |
|  | (0.096) | (0.096) | (0.110) | (0.094) |

*Note:* This table presents the AI treatment effects across languages on participants’ self-reported perceived difficulty about the email-writing task. Difficulty is operationalized on a 1-5 Likert scale (1 = very easy; 5= very difficult). Standard errors clustered at the participant-level in parentheses. *p<0.1; **p<0.05; ***p<0.01.

**Table S13. Effects of Language on Email Quality, Productivity, and Confidence by Task Type**

|  |  | | | | | | | | |
| --- | --- | --- | --- | --- | --- | --- | --- | --- | --- |
|  |  | | | | | | | | |
|  | *Completeness*  (1) | *Relevance*  (2) | *Actionability*  (3) | *Creativity*  (4) | *Length*  (5) | *Duration*  (6) | *Confidence*  (7) | *Difficulty*  (8) |  |
|  | | | | | | | | | |
| AI Treatment | 0.112 | 0.250 | 0.500 | 0.556 | 275.425 | 76.488 | -0.362 | 0.237 |  |
|  | (0.298) | (0.302) | (0.335) | (0.353) | (252.910) | (161.641) | (0.256) | (0.298) |  |
| Arabic | -0.123 | 0.260 | -0.077 | -0.196 | -307.573 | 381.588 | 0.123 | -0.085 |  |
|  | (0.333) | (0.323) | (0.309) | (0.365) | (189.256) | (400.988) | (0.174) | (0.243) |  |
| Chinese | -0.144 | 0.403 | -0.028 | 0.039 | -378.672^**^ | 132.828 | -0.744^***^ | 0.578^**^ |  |
|  | (0.277) | (0.280) | (0.298) | (0.306) | (164.946) | (153.892) | (0.245) | (0.242) |  |
| Customer Service | 0.474 | 0.636^**^ | 0.239 | 0.085 | -78.189 | -73.254 | -0.278 | 0.039 |  |
|  | (0.290) | (0.265) | (0.312) | (0.310) | (174.714) | (92.349) | (0.253) | (0.219) |  |
| Human Resources | 0.300 | 0.687^***^ | 0.406 | 0.431 | -208.137 | 8.300 | -0.488^*^ | 0.425^**^ |  |
|  | (0.269) | (0.247) | (0.289) | (0.318) | (189.167) | (116.175) | (0.283) | (0.206) |  |
| R&D | -0.422 | -0.125 | -0.389 | -0.517 | -412.839^***^ | -19.728 | -0.356 | 0.744^**^ |  |
|  | (0.295) | (0.293) | (0.311) | (0.330) | (157.303) | (156.647) | (0.268) | (0.317) |  |
| AI Treatment × Arabic | 0.453 | 0.437 | -0.102 | -0.246 | 95.148 | -568.383 | 0.082 | 0.147 |  |
|  | (0.492) | (0.459) | (0.435) | (0.529) | (355.286) | (432.488) | (0.315) | (0.431) |  |
| AI Treatment × Chinese | 0.332 | -0.072 | -0.119 | -0.092 | 362.691 | -165.148 | 0.895^**^ | -0.633 |  |
|  | (0.448) | (0.452) | (0.425) | (0.453) | (325.464) | (233.626) | (0.362) | (0.422) |  |
| AI Treatment × Customer Service | 0.071 | -0.035 | 0.189 | 0.152 | 50.171 | 432.626^*^ | 0.365 | -0.215 |  |
|  | (0.425) | (0.401) | (0.430) | (0.464) | (333.492) | (235.396) | (0.401) | (0.359) |  |
| AI Treatment × Human Resources | 0.238 | -0.137 | 0.019 | -0.012 | 535.063 | 79.262 | 0.700 | -0.612 |  |
|  | (0.388) | (0.384) | (0.410) | (0.458) | (381.891) | (234.398) | (0.425) | (0.382) |  |
| AI Treatment × R&D | 1.426^***^ | 1.145^***^ | 1.284^***^ | 1.558^***^ | 871.069^***^ | 60.448 | 0.392 | -0.261 |  |
|  | (0.375) | (0.396) | (0.418) | (0.440) | (327.392) | (237.899) | (0.410) | (0.453) |  |
| Arabic × Customer Service | -0.077 | 0.006 | 0.259 | 0.103 | 177.670 | 33.190 | 0.618^**^ | 0.082 |  |
|  | (0.457) | (0.448) | (0.447) | (0.461) | (251.391) | (472.915) | (0.313) | (0.346) |  |
| Chinese × Customer Service | -0.363 | -0.449 | -0.211 | -0.355 | 55.011 | -84.523 | 0.318 | -0.031 |  |
|  | (0.399) | (0.408) | (0.428) | (0.406) | (230.104) | (185.088) | (0.390) | (0.330) |  |
| Arabic × Human Resources | -0.158 | -0.541 | -0.548 | -0.523 | 219.767 | -193.401 | 0.689^**^ | -0.540 |  |
|  | (0.493) | (0.434) | (0.428) | (0.486) | (289.901) | (460.394) | (0.343) | (0.352) |  |
| Chinese × Human Resources | 0.219 | -0.140 | 0.297 | -0.095 | 441.020^*^ | 127.822 | 0.382 | -0.703^**^ |  |
|  | (0.368) | (0.370) | (0.380) | (0.410) | (236.410) | (241.246) | (0.394) | (0.325) |  |
| Arabic × R&D | 0.068 | -0.204 | 0.105 | 0.057 | 252.601 | -155.977 | 0.432 | -0.026 |  |
|  | (0.488) | (0.470) | (0.473) | (0.509) | (242.099) | (457.098) | (0.351) | (0.422) |  |
| Chinese × R&D | 0.117 | -0.528 | 0.135 | 0.034 | 589.686^**^ | 68.387 | 0.488 | -0.397 |  |
|  | (0.436) | (0.476) | (0.430) | (0.441) | (238.976) | (251.679) | (0.399) | (0.425) |  |
| AI Treatment × Arabic × Customer Service | -0.072 | -0.255 | -0.393 | 0.042 | -152.233 | -60.281 | -0.655 | 0.170 |  |
|  | (0.701) | (0.603) | (0.622) | (0.698) | (472.375) | (552.382) | (0.539) | (0.567) |  |
| AI Treatment × Chinese × Customer Service | 0.518 | 0.367 | 0.105 | -0.010 | 4.012 | -323.266 | -0.692 | 0.024 |  |
|  | (0.611) | (0.628) | (0.575) | (0.585) | (430.594) | (313.062) | (0.558) | (0.529) |  |
| AI Treatment × Arabic × Human Resources | -0.189 | -0.081 | -0.073 | 0.140 | -685.017 | 530.029 | -0.295 | 0.395 |  |
|  | (0.711) | (0.619) | (0.629) | (0.710) | (509.498) | (559.791) | (0.541) | (0.609) |  |
| AI Treatment × Chinese × Human Resources | -0.340 | 0.357 | -0.463 | -0.149 | -872.194^*^ | -180.669 | -0.683 | 0.952^*^ |  |
|  | (0.573) | (0.567) | (0.535) | (0.594) | (464.920) | (370.859) | (0.572) | (0.545) |  |
| AI Treatment × Arabic × R&D | -0.991 | -0.915 | -1.238^*^ | -0.646 | -931.626^**^ | 385.503 | -0.445 | -0.346 |  |
|  | (0.658) | (0.618) | (0.636) | (0.682) | (444.343) | (514.301) | (0.529) | (0.622) |  |
| AI Treatment × Chinese × R&D | -0.679 | 0.037 | -0.883 | -1.340^**^ | -1,028.975^**^ | -95.696 | -0.877 | -0.028 |  |
|  | (0.612) | (0.653) | (0.584) | (0.593) | (439.305) | (340.182) | (0.571) | (0.642) |  |
| Constant | 3.200^***^ | 3.125^***^ | 3.000^***^ | 2.850^***^ | 1,443.450^***^ | 1,379.950^***^ | 3.800^***^ | 2.700^***^ |  |
|  | (0.214) | (0.196) | (0.241) | (0.244) | (138.980) | (65.785) | (0.156) | (0.164) |  |

*Note:* This table presents the AI treatment effects across languages with task type as an interaction term. The baseline language group is “English,” and the baseline task type is “Marketing.” Standard errors clustered at the participant-level in parentheses. *p<0.1; **p<0.05; ***p<0.

**References**

1. Zahra, S. A. & George, G. Absorptive capacity: A review, reconceptualization, and extension. *Academy of Management Review* **27**, 185–203 (2002).

2. Camisón, C. & Forés, B. Knowledge absorptive capacity: New insights for its conceptualization and measurement. Journal of Business Research. *J Bus Res* **63**, 707–715 (2010).

3. Legendre, P. Species associations: the Kendall coefficient of concordance revisited. *J Agric Biol Environ Stat* **10**, 226–245 (2005).

4. Siegel, S. & Castellan, N. J. , Jr. *Nonparametric Statistics for the Behavioral Sciences*. (McGraw-Hill, New York, 1988).
